# Statistical Emulation of Neural Simulators: Application to Neocortical L2/3 Large Basket Cells

**DOI:** 10.3389/fdata.2022.789962

**Published:** 2022-03-25

**Authors:** Gilad Shapira, Mira Marcus-Kalish, Oren Amsalem, Werner Van Geit, Idan Segev, David M. Steinberg

**Affiliations:** ^1^Department of Statistics and Operations Research, Tel Aviv University, Tel Aviv, Israel; ^2^Division of Endocrinology, Beth Israel Deaconess Medical Center, Harvard Medical School, Harvard University, Boston, MA, United States; ^3^Blue Brain Project, École Polytechnique Fédérale de Lausanne (EPFL), Campus Biotech, Geneva, Switzerland; ^4^The Edmond and Lily Safra Center for Brain Sciences, The Hebrew University of Jerusalem, Jerusalem, Israel

**Keywords:** emulator, Gaussian process, random forest, *in silico* experiment, neural network, NEURON simulator

## Abstract

Many scientific systems are studied using computer codes that simulate the phenomena of interest. Computer simulation enables scientists to study a broad range of possible conditions, generating large quantities of data at a faster rate than the laboratory. Computer models are widespread in neuroscience, where they are used to mimic brain function at different levels. These models offer a variety of new possibilities for the neuroscientist, but also numerous challenges, such as: where to sample the input space for the simulator, how to make sense of the data that is generated, and how to estimate unknown parameters in the model. Statistical emulation can be a valuable complement to simulator-based research. Emulators are able to mimic the simulator, often with a much smaller computational burden and they are especially valuable for parameter estimation, which may require many simulator evaluations. This work compares different statistical models that address these challenges, and applies them to simulations of neocortical L2/3 large basket cells, created and run with the NEURON simulator in the context of the European Human Brain Project. The novelty of our approach is the use of fast empirical emulators, which have the ability to accelerate the optimization process for the simulator and to identify which inputs (in this case, different membrane ion channels) are most influential in affecting simulated features. These contributions are complementary, as knowledge of the important features can further improve the optimization process. Subsequent research, conducted after the process is completed, will gain efficiency by focusing on these inputs.

## Introduction

Many systems in the physical, biological, and engineering sciences are now studied by running computer codes that simulate generation of data, mathematically mimicking the natural phenomenon. There are many reasons why computer simulation may be the preferred platform for investigation. Some systems cannot be studied by traditional laboratory experiments, perhaps due to the high costs of the laboratory experiment, the limited availability of experimental samples, or because laboratory experimentation of the system is simply infeasible. Simulation experiments (known in the life sciences as *in silico* experiments and in statistical parlance as computer experiments) can explore behavior over a variety of input settings.

*In silico* experiments present new possibilities (and challenges) when compared to traditional laboratory experiments, but there are also many similarities: what are the factors of the experiment, which will serve as control factors at constant levels and which will be varied and investigated, what combinations of factor levels should be used in the experiment, and more. This work will focus on new features that arise when the laboratory is replaced by a computer as the main experimental tool, and especially on the new challenges these unique features present for statistical modeling. It breaks new ground in showing how these modeling efforts can enhance the optimization of neural simulators and by proposing methods appropriate for features that never occur in the simulated output for some input settings.

### *In silico* Experiments in Neuroscience

Neuroscience is a domain where experiments may be too expensive, too risky, and sometimes impossible, to perform in a lab. Consequently, there is great interest in research to simulate the brain at a variety of levels and scales. Makin (2019) concisely summarizes four major challenges: scale, complexity, speed, and integration, describing major progress to date and expectations for the near future.

The examples below illustrate the use and value of *in silico* models in recent neuroscience research.

#### Deep Brain Stimulation

Deep brain stimulation (DBS) is a surgical procedure used mainly to treat movement disorders. The stimulation is achieved by applying an electric pulse to a pre-specified target location in the brain. A critical part of this procedure is the adjustment, a-priori, of the electrical parameters of the DBS device, such as the amplitude and pulse width (Perlmutter and Mink, 2006).

Thousands of different parameter combinations are available, so finding a successful combination for a specific patient may be long and dangerous. Instead, computer models have been developed that can be adapted to the specific patient to predict the neurostimulation effect of an electrical parameter combination. The current “gold standard” method is to couple electric field data to multi-compartment neuron models, which are based on the structure of the neuron and on the solutions of differential equations for the plasma membrane and the membrane potential (McNeal, 1976; Chaturvedi et al., 2013; Pava et al., 2015).

#### Brain Simulation Platform and Multiscale Modeling

The Brain Simulation Platform (BSP) of the European Human Brain Project (HBP) aims to develop a computerized replica of the human (and other animals) brain, and its dynamics. There are several goals for the BSP: first, to enable researchers to conduct investigations that are not possible in a lab; second, to offer a similar approach to lab experiments, and thus reduce the need for animal brain experiments; third, to offer researchers a method to compare their experimental results with computer model predictions.

### Statistical Modeling and Analysis of Computer Experiments

The increased use of simulation platforms for research has been accompanied by an extensive body of statistical research on effective approaches to model and analyze the data that they generate (Levy and Steinberg, 2010; Santner et al., 2018).

There are two main benefits to this statistical modeling. First, many computer experiments rely on complex simulators involving numerical solvers of the underlying equations. One run of the simulator may require substantial resources (including time), so that the investigation of all desired combinations of factor levels may exceed available resources. Statistical modeling can provide an emulator, or surrogate model, of the simulator. With the emulator, much more extensive exploration of factor settings is possible. This consideration is especially important in applications where the goal is to optimize a system, which may require many function calls.

Second, the fit and analysis of a statistical model can identify the input factors with the strongest effects on the output variable(s). Although the role of each input is known from the work that went into building the simulation model, many simulators are so complex that it is difficult to determine from first principles which factors are most important. In addition, analysis can be essential for uncertainty quantification, which aims to understand output variation when there is large uncertainty regarding input values.

Following the example of DBS in the preceding section (DBS), according to Chaturvedi et al. (2013), the McNeal simulation method mentioned above is too slow (due to its computational burden) to be practical for most clinical applications. Therefore, simplified, fast and accurate methods are needed. Pava et al. (2015) developed a new methodology to reduce the computation time required to estimate the volume of tissue activated during deep brain stimulation. At the heart of their method is an emulator that replaces the system of differential equations with combined multi-compartment axon models coupled to the stimulating electric field by a Gaussian process classifier estimated from outcome data. The approach of Pava et al. (2015) reduced by a factor of 10 the average computational runtime of Volume Tissue Activated estimation, while maintaining a prediction error rate below 20%.

Statistical modeling is also important when a simulator is inherently stochastic. Papamakarios et al. (2019) showed how such models can be used to estimate the consequent density function of observed data and thus provide a sound basis for statistical inference. Lueckmann et al. (2017) took this idea and adapted it for use with neural simulation models. Cranmer et al. (2020) provides an excellent overview of several related approaches to model-driven inference for simulation models.

### Outline

Our goal in this work is to show how statistical emulation of complex computer simulations can be effectively used in neuroscience. The focus is on a particular use case involving optimization of parameters for a model developed and run in the NEURON environment. The next section of the paper gives a brief summary of the statistical ideas and methods that were applied. We then describe the simulation model and the optimization task. The following section presents the results and a brief summary followed by Discussion and Conclusions.

## Brain Simulations

### NEURON Simulations

NEURON (Hines, 1984; Hines and Carnevale, 1997) is a popular and flexible simulation environment for implementing biologically realistic models of electrical and chemical signaling in neurons and networks of neurons. The simulator describes the unique spread and interaction of electrical and chemical signals within and among neurons in different parts of the brain. It is based on a constrained non-linear equation system that does not have an analytical solution. NEURON provides an efficient numerical solution, enabling researchers to illustrate and explore the operation of brain mechanisms, cross-validate their data, estimate experimental parameters, test hypotheses, and perform robust and groundbreaking experiments that are difficult, if not infeasible, to perform in a lab due to technical difficulties as well as ethical limitations.

NEURON was designed specifically to simulate the equations that describe the electrical and chemical activity of nerve cells, in order to offer a flexible framework for handling problems in which membrane currents are complex, and spatially inhomogeneous. The outputs of the simulator are step by step evolutions of electrical and chemical values of a cell. The input parameters of this paper's use case are membrane ion channels whose opening/closing in response to voltage and/or a chemical is the basis for the electrical activity of neurons.

NEURON simulations can also serve as the empirical basis for biologically realistic quantitative models that formulate new hypotheses of brain function. Some examples of topics that were investigated with such simulation-based models are given in Hines and Carnevale (1997), and the list has grown significantly during recent years.

### Multiple Objective Optimization

The novel framework of multiple objective optimization (MOO) was developed using the NEURON simulator for automatically constraining parameters (in particular of the membrane ion channels) in compartmental models of neurons, given a large set of experimentally measured responses of these neurons (Druckmann et al., 2007). This method accounts for the variability of experimental voltage responses, even for the exact same input, and provides several error functions that characterize the difference (in experimental standard deviation units) between experimental voltage traces and simulated model responses, and can be performed on the basis of individual features of interest (e.g., spike rate, spike width). Druckmann et al. (2007) consolidated their method with a genetic search algorithm and showed an excellent fit between model behavior and the laboratory firing pattern of two distinct electrical classes of cortical interneurons, accommodating and fast-spiking. The process has important implications, as these cells serve as building blocks for more complex neural networks and the exact channel densities affect the network behavior (Egger et al., 2014; Markram et al., 2015; Casali et al., 2019; Amsalem et al., 2020; Billeh et al., 2020).

#### Framework

Every fitting attempt between model performance and experimental data with this method is based on an experimental target dataset (and the stimuli that generated it), a simulator with corresponding parameters (and their ranges), and a search method. The result of the fitting procedure is a solution (or sometimes a set of solutions) of the best fitting parameter values as quantified by a score function that accounts for the distance between the model output and the target experimental data. At the heart of the score function is a sum of objective scores for each set of parameters, or “individual,” where each objective score is normalized into units of experimental standard deviations: $\frac{{f_{sim}-\mu }_{exp}}{\sigma_{exp}}$, where *f*_*sim*_ is the simulated objective value, and μ_*exp*_, σ_*exp*_ are the mean and standard deviation of the feature values from the laboratory experiment. After an objective score is calculated for each feature *j*, the scores are summed into a unified global score:


(1) $$\text{Global}\;\text{score}\;\left(\text{individual}\right)=\sum_{j}^{}\;\left|\frac{{\text{f}}_{\text{j},\text{sim}}(\text{individual})-{\text{μ}}_{\text{j},\text{exp}}}{{\text{σ}}_{\text{j},\text{exp}}}\right|.\text{​​​}$$

#### Genetic Algorithm

To find good individuals, Druckmann et al. (2007) used a genetic algorithm (GA) that had proved effective for constraining conductance-based compartmental models. The main idea behind a genetic algorithm (GA), introduced by Holland in 1960 and developed by Goldberg (1989), is to solve optimization problems with a population of candidate solutions that evolves simultaneously toward the optimal solution. The evolution starts from a set of *n* randomly drawn solutions that cover the entire search space. The score function is calculated for each selected individual and individuals with high scores are selected as “parents” to produce the next set of solutions, or “children.” That process involves both fixed rules for combining existing individuals and a number of stochastic elements. The former direct the GA to more successful individuals in later generations, while the latter force the GA to explore the space and limit the risk of converging to a local optimum. The process is then repeated using the newly derived children as the parents for the next generation.

In general, the combination of MOO and GA can be described by the following flowchart.

### Use Case

This paper explores the simulated data researched in the work of Amsalem et al. (2016) on neocortical L2/3 large basket cells (LBC). This study utilized a feature-based multi-objective optimization (MOO) protocol (Druckmann et al., 2007; Ramaswamy and Markram, 2015) to fit the isolated L2/3 LBC neuron model to *in vitro* voltage traces. The experimental voltage traces used as a target for the model consisted of 4 different stimulus protocols, two of which are studied here: (1) Three different subthreshold current injections (−0.22, 0.04, and 0.15 nA) of 1,000 ms each (IVf_0, IVf_3, IVf_5); (2) Five repetitions of long (2,000 ms) suprathreshold current injections (0.27 nA) (IDRest_7). All simulations were performed in the NEURON simulator (Hines and Carnevale, 2008) running both on local clusters (NEURON 7.3) and on a supercomputer (NEURON 7.4).

The free parameters in the optimization were the specific membrane resistivity, the densities of 11 active ion channels, and the dynamics of intracellular Ca^2+^ (Hay et al., 2011). The neuron was separated into different regions: (1) the axon initial segment, (2) the soma, and (3) the dendrites. Each region had a separate set of membrane channels with different conductance densities. The full list of parameters for each region is provided in Appendix A.

The 28 electrical features used as outputs in this simulation were basic features (such as input resistance, spike shape, and frequency). The full list of these features is provided in Appendix B. These features were extracted from the electrical trace produced by the simulator for a given stimulus, and compared to the corresponding feature values for all experimental repetitions of that stimulus (that were extracted from the experimental trace) for the same given stimulus.

The GA was run for 198 generations, each containing 1,000 individuals (consisting of 31 parameters each) and the multivariate output of 28 electrical features for each individual. Lists of all parameters and features, including their mean and standard deviation laboratory values, can be found in Appendices A, B, respectively.

The BluePyOpt Python package (Van Geit et al., 2016) was used to activate the NEURON simulator, receive scores for each individual in the generation, and send them to DEAP (De Rainville et al., 2012; Fortin et al., 2012), a Python library that implements GA procedures.

## Statistical Methods

This section describes the strategy underlying the implementation of statistical emulators, the specific emulation methods that were assessed, and for some of the methods, associated ideas for statistical inference.

The general problem of emulation has the following elements. Given a vector ***x*** of inputs, and a simulator that generates output ***f***(***x***), we want to construct an empirical model, or *emulator*, $\hat{f}\left(x\right)$. The output could be a function (e.g., an electrical time trace), a scalar (e.g., a feature like the time to first spike), or a vector of features. The inputs could be experimental conditions (e.g., an external current) or values of unknown simulator parameters (e.g., conductance of different cells). The simulator output is observed on a training sample of input vectors and the training data are used to fit the emulator.

### Emulation Strategy

**Figure 1:**
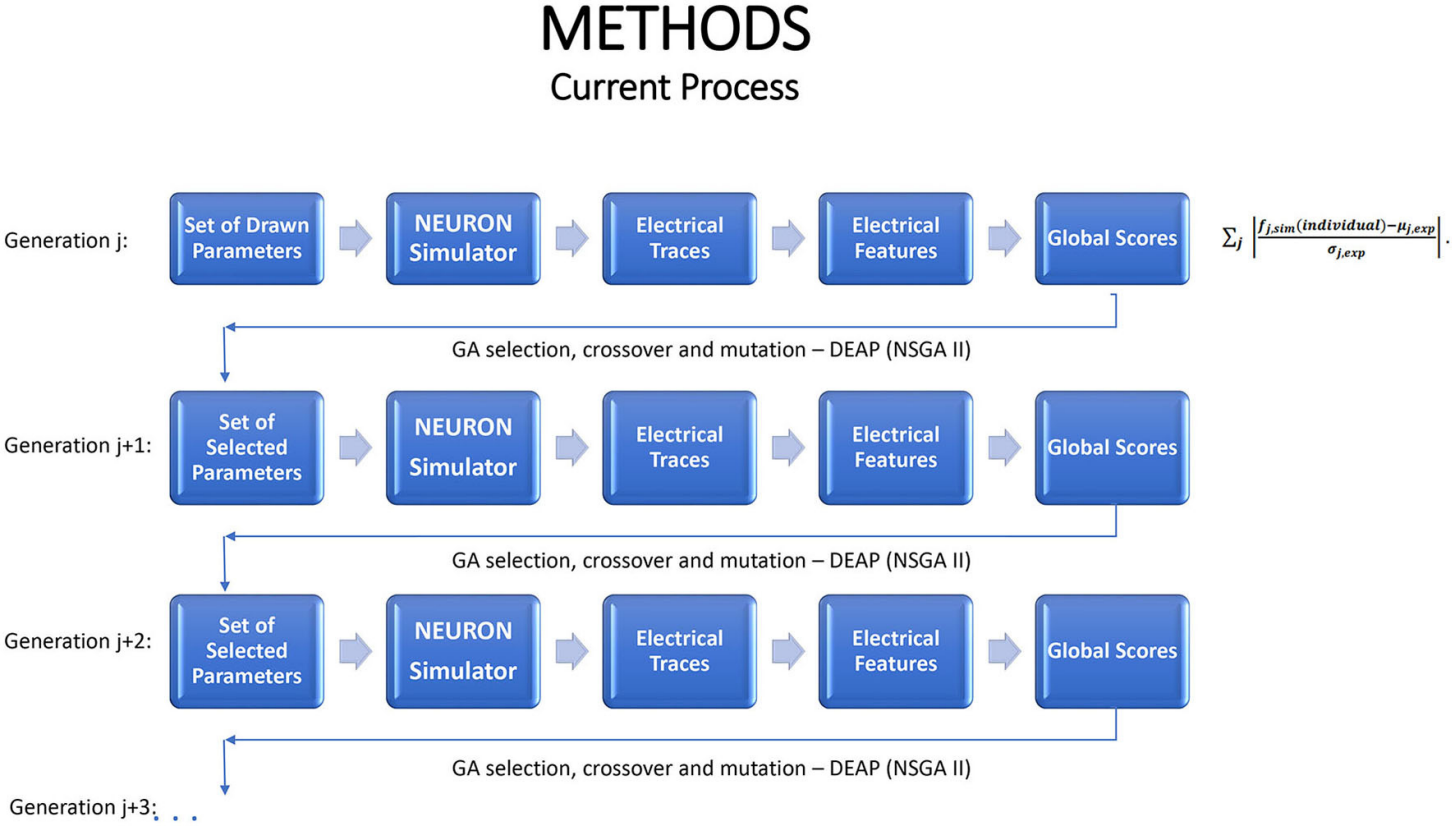
A schematic portrayal of how the genetic algorithm advances from one generation to the next, when used for multiple objective optimization in conjunction with the NEURON simulator. Each generation should lead to increasingly favorable individuals.

We exploit emulation as a tool in working with the NEURON simulator to perform multi-objective optimization (MOO). As shown in Figure 1, in the GA optimization process the simulator functions as a steppingstone to each new generation. The simulator runs require substantial computational resources, provided by a large cluster; the process is not feasible on a single computer. Even on the cluster, the average run time is ~1 h for each generation and the entire optimization process lasts dozens and even hundreds of hours. With such resource-heavy simulators, there is good potential to benefit by replacing, or supplementing, the simulator by an emulator.

The simulator runs used to train the emulators are taken from the GA iterations used in carrying out MOO. This supports a “proof of concept” analysis in which we can show that emulators can effectively complement the NEURON simulator. We describe emulator fits at several different stages of the GA iterations.

The first generation of the GA optimization process simulates output at a random sample of n input vectors (“parents”). This sample covers the entire input space and so provides a basis for emulating global input-output relationships. The results of this modeling strategy will be presented in the section on Emulation of Selected Electrical Features. Subsequent GA iterations are expected to concentrate samples in “promising” regions of the input space, with few samples in regions that do not match the laboratory data. We expect that the global emulators from the initial iteration will begin to lose their predictive ability as the sampled inputs become more concentrated. Thus, it is desirable to periodically refit emulators to samples from the most recent GA generations.

**Figure 2:**
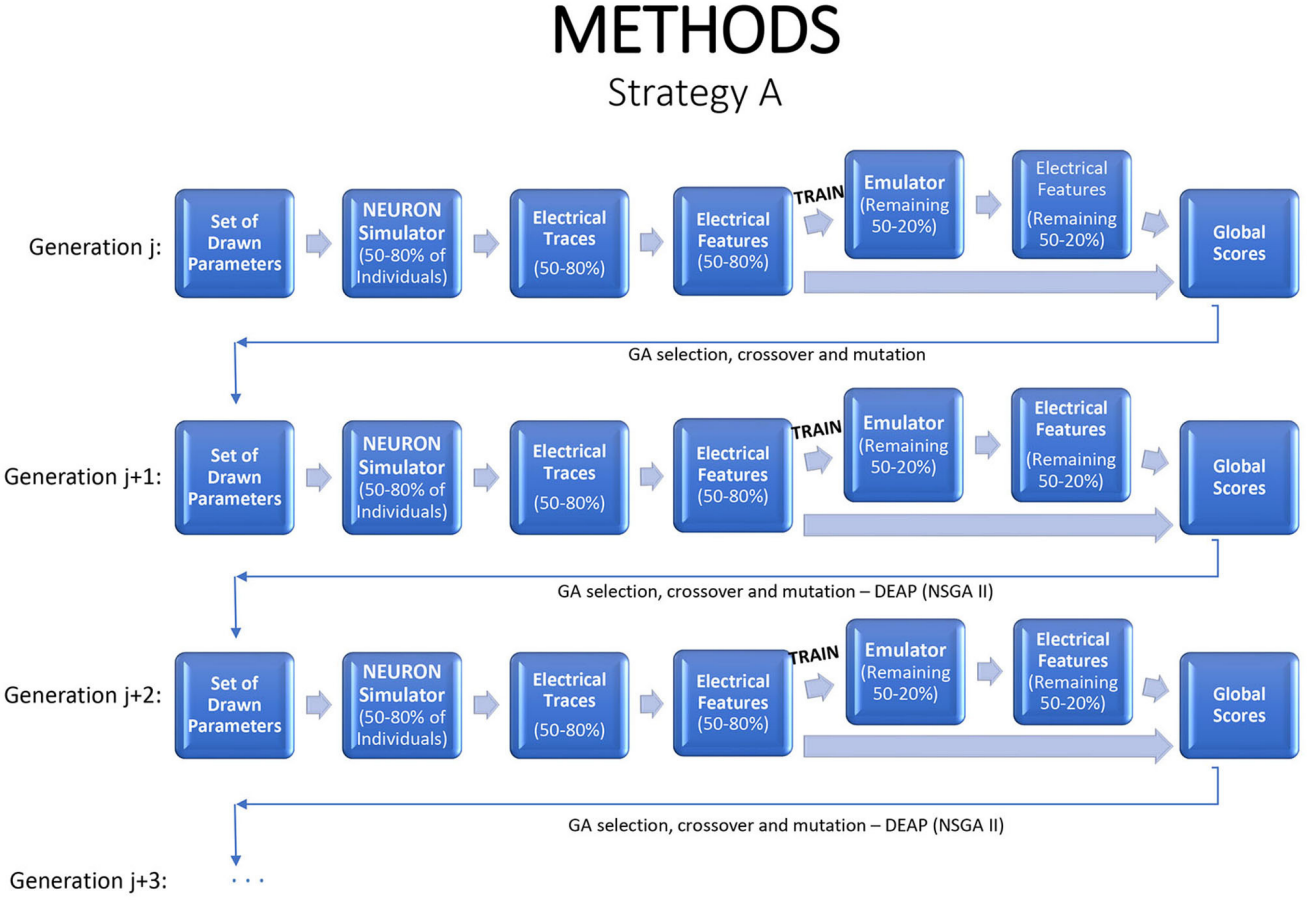
A strategy for a modified genetic algorithm, simulating features for 80% of the individuals, using them to fit an emulator, and replacing the simulator runs by emulator predictions for the remaining 20%.

Emulation can be injected into the iterative GA optimization process in a number of different ways. If an emulator can be tuned quickly, one option is to refit it at each generation, with the simulator applied to some fraction of the new children to generate training data, and the revised emulator used to evaluate or to screen the remaining children. This strategy is illustrated in Figure 2, with 80% of the individuals used to train the emulator, which is then applied on the remaining 20% to predict their electrical features. This strategy saves 20% of the simulator run time and fitting the emulator permits deeper exploration of the parameter space and statistical inference. If the emulators are highly accurate, the fraction of training data can be reduced.

**Figure 3:**
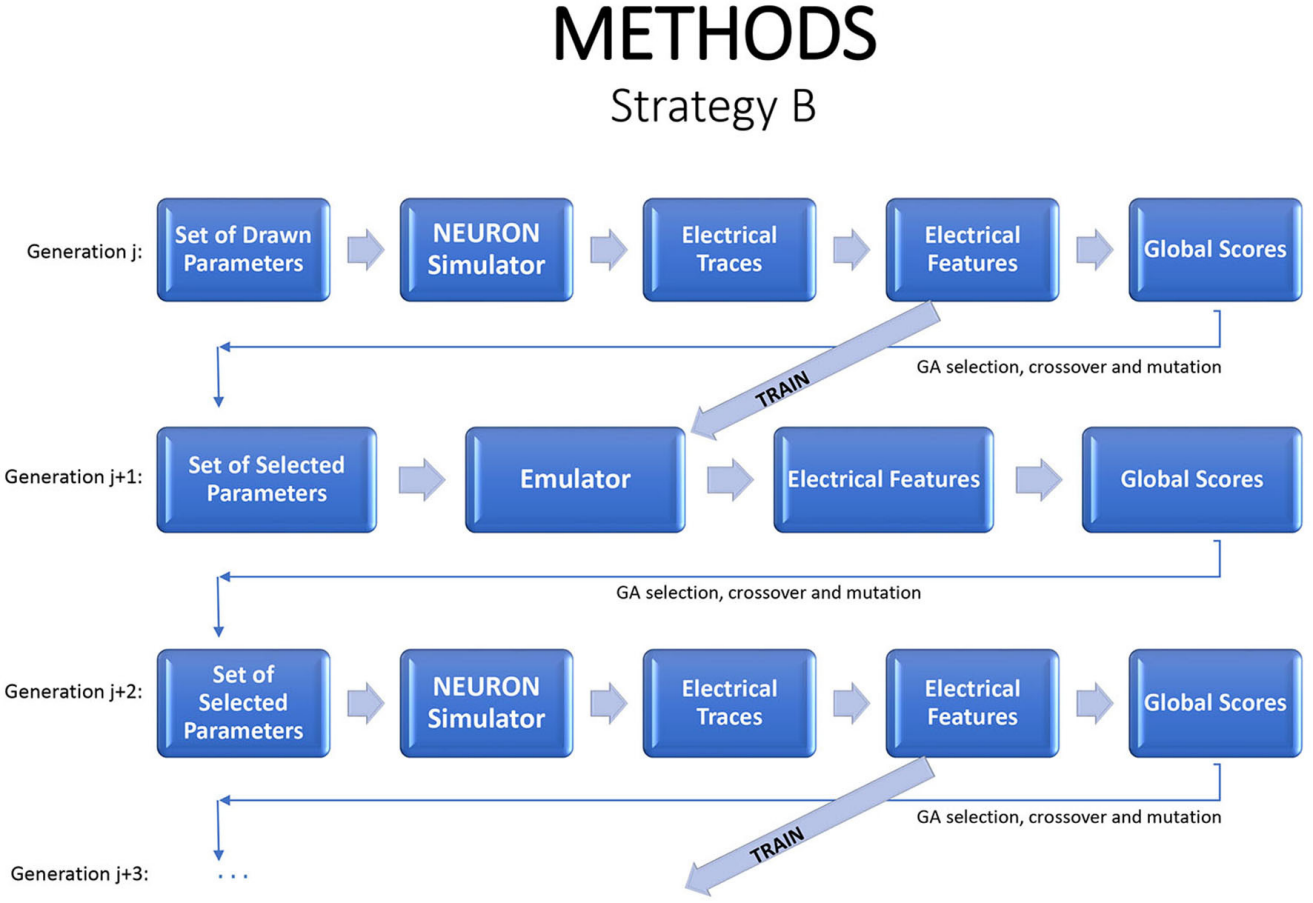
A strategy for a modified genetic algorithm, simulating the individuals in one generation, using them to fit an emulator, and replacing the simulator runs by emulator predictions, for the following generation. Periodically a new generation should be evaluated by the simulator in order to update the emulator.

An alternative strategy is to produce new generations entirely from the emulator. One option is to evaluate a generation with the simulator, to use those results to estimate an emulator, and then to evaluate subsequent generations with the emulator. As noted earlier, due to the natural drift of the genetic algorithm in parameter space, it will usually be necessary to return to the simulator periodically, generating new training data and fitting a new emulator. In each generation, one can run the simulator on a small sample of children to check if the emulator continues to accurately mimic the simulator. This strategy can dramatically reduce the simulation budget. Figure 3 illustrates the flow.

An intermediate approach is to use the emulator from one generation to “screen” children that are proposed at the next generation. The GA may create offspring that are not at all successful. Emulating the outcomes for the offspring can often highlight these poor performers among the offspring, eliminating the need to assess them *via* full simulation.

A more ambitious strategy is to replace the GA entirely, using optimization strategies in which the emulator replaces the simulator in iterative objective function evaluations. See, for example, Taddy et al. (2009), Yao et al. (2014), Eames et al. (2015), Springenberg et al. (2016) and Bischl et al. (2017). This more ambitious strategy was not implemented in the current use case and will be considered in future work. Acerbi and Ma (2017) applied emulation methods to a complex likelihood resulting from a neural model for visual perception. Their approach uses Gaussian processes and exploits a Bayesian approach that approximates the objective function and provides uncertainty measures that guide where to make further function evaluations.

### Emulation Methods

Emulators can be produced using many prediction methods that have been proposed in the statistics and machine learning literature. The following sections briefly review the statistical methods and models used here. These models have a number of attractive features for modeling data from computer experiments (Rasmussen and Williams, 2006; Ghahramani, 2013).

#### Gaussian Process Regression

Gaussian process regression (GPR) models were first proposed in geostatistics (Krige, 1951) as a method for spatial interpolation of two or three-dimensional data. They are very flexible models and, following Sacks et al. (1989), they have been a popular choice in the statistical analysis of computer experiments. They have also become an important tool in machine learning (Rasmussen and Williams, 2006; Ghahramani, 2013). GPR can be used directly to emulate a numerical output. For classification problems, it can be used to estimate the probability of a given class occurring for a given input location.

As in Sacks et al. (1989), view the output ***f***(***x***)as the realization of a random process that includes a regression model and a stochastic component ***Z***(***x***) that is a Gaussian process with mean 0 at all ***x***:


(2) $$\text{f}\left(\text{x}\right)=\;\sum_{\text{i}=1}^{\text{n}}{\text{β}}_{\text{i}}{\text{g}}_{\text{i}}(\text{x})+\text{Z}(\text{x}).$$

where the ***g***_*i*_(***x***) are known regression functions, the **β*_i_*** are unknown coefficients and ***Z***(***x***) represents the deviation of the simulator from the regression model. Welch et al. (1992) advised removing all regression terms except a constant, so that all the dependence on ***x*** is reflected *via* ***Z***(***x***). Rasmussen and Williams (2006) took a further step, advocating models with no regression terms at all.

One of the attractive properties of the GPR emulator is that it interpolates the training data: if ***x*** is one of the observation sites, then $\hat{f}\left(x\right)=f\left(x\right)$. This is very appealing when the training data are observed with no random error, as often happens with neuroscience simulators.

An essential element of the method is the covariance function, or “kernel,” *k*(*x, x*′) of ***Z***(***x***). The covariance function describes the similarity of the output at different input settings and, not surprisingly, emulated values rely primarily on those training data highly correlated with the emulation site.

One common option is to normalize each input variable and then apply the squared exponential family:


(3) $$\begin{array}{l} k_{y}\left({\text{x}}_{p},{\text{x}}_{q}\right)=\sigma_{f}^{2}exp\left(-\frac{1}{2l^{2}}{|{\text{x}}_{p}-{\text{x}}_{q}|\;}^{2}\right). \end{array}$$

The hyperparameter $\sigma_{f}^{2}$ is often called signal variance, and *l* is the length scale. Often each factor is given a distinct length scale *l*_*j*_, with large values indicating important factors. The formulation can be expanded to handle noisy training observations (i.e., stochastic simulators). A number of efficient methods have been proposed for estimating the hyperparameters. In practice, most emulators then condition on these estimated values (in the spirit of empirical Bayes). There are also fully Bayesian approaches that average the emulator with respect to the posterior uncertainty in the hyperparameters (Harari and Steinberg, 2013; Pronzato and Rendas, 2017).

One drawback to GPR is computational complexity, especially for large data sets, since GP models require inversion of *N*×*N* covariance matrices, where *N* represents the number of desired evaluation and prediction points, and the computation time of the standard method for computer matrix inversion of positive definite symmetric *N*×*N* matrices, Gauss–Jordan elimination, is *O*(*N*^3^).

Thus, some simplified methods have also been proposed. One option is to enable a data-driven choice, for each input factor, as to whether its relationship to the output will be linear or GP in form (Gramacy and Lee, 2008). This is known as the Gaussian Process limiting linear model, or GPllm. Another is to use only a subset of the training data in making a prediction—known as local approximate Gaussian Process (laGP) (Gramacy and Apley, 2014; Park and Apley, 2018). The subset is chosen in an optimal manner on the basis of the assumed kernel.

#### Random Forest

The random forest (Breiman, 2001) is a tree-based method for non-parametric prediction or classification. Treed partitions divide up the input space recursively, using binary splits of the data on the value of a single variable. The prototypical treed partition is Classification and Regression Trees (CART) (Breiman et al., 1984), which is very easy to use and interpret. The prediction for a new input vector is the average of the training cases in the same partition. Classifications are assigned by majority vote within the partition. CART uses all the training data to generate a single prediction/classification model. Often this leads to a tree that over-emphasizes peculiar features in the data. Random forests address this drawback by building a collection of trees and introducing some randomness into the process, both in terms of the training cases used for each binary split and the features that are candidates for splitting. The resulting predictor for an input vector *x* is the average prediction over all the trees in the forest. The random forest classifier for *x* is the class assigned by the largest number of trees. Random forest is considered an excellent “all purpose” prediction/classification method (Efron and Hastie, 2016, Chapter 17).

#### Bayesian Treed Models and Treed Gaussian Process Regression

Chipman et al. (2002) proposed a Bayesian approach to finding and fitting treed models. The method assigns a prior distribution to the tree structure that prefers smaller trees and larger partitions and then guides a Markov Chain Monte Carlo (MCMC) search toward “more promising” treed models, thereby fully exploring the model space. The resulting posterior includes a collection of trees sampled by the MCMC, each with a prediction model (typically a simple regression model) conditioned on the tree. The priors and posterior estimation techniques proposed by Chipman et al. (2002) are summarized in the Supplementary Materials.

Gramacy and Lee (2008) and Taddy et al. (2009) combined the Bayesian treed models (Chipman et al., 2002) with GPR, leading to Treed Gaussian Process (TGP) models. The TGP follows Chipman et al. (2002) in its treatment of tree structure, but replaces the simple regression for each partition with a GPR model. The TGP thus has two mechanisms for capturing complex input-output relationships: like other treed models, it can adapt to changes in relationships across the input space; and in each partition, it has the modeling flexibility of GPR. Gramacy and Lee (2008) noted that simple models might be fine for some partitions. They added binary variables that indicate, for each input factor and within each partition, whether the final prediction model is linear or GPR. Each MCMC iteration makes a new draw of these indicators. The linear model can be expressed as a limit of GPR, so they referred to this as a limiting linear model (LLM) and to the overall model as TGP-LLM.

#### Neural Networks

A neural network is a highly parametrized model considered as a “universal approximator”—meaning it is a model that, with enough data, could learn any smooth predictive relationship. The original purpose of NN models was to model the neural networks found in the human brain, hence its name and network architecture. Readers interested in more details regarding NN models can view (McCulloch and Pitts, 1943; Efron and Hastie, 2016, Chapter 18;).

### Emulation Sensitivity Analysis

One of the benefits of fitting an emulator is that it can point to the input factors with the largest (or smallest) effects on the output(s). This is known as “sensitivity analysis” and was introduced by Saltelli (2002). Saltelli proposed sensitivity analysis for use directly with simulator output, but it is equally useful when applied with an emulator, with the advantage that it can be much faster to compute.

The focus is on how predictive means and variances change when fixing some input coordinates and integrating out others. For example, one might want to know what is the typical time to first spike for a particular conductance is. As this time also depends on other model inputs, the idea is to fix the conductance of interest and average over the others, with respect to a distribution that reflects either best knowledge of their likely values (for example, a posterior density) or relative interest in different settings. When working with an emulator that provides only a point prediction, sensitivity analysis is limited to assessing such typical value dependence. For emulators like GPs, which give a posterior distribution, other aspects of the distribution (e.g., the variance, or some quantiles) can also be averaged.

## Results

### Emulation of Selected Electrical Features

This section provides a “proof of concept” presentation of the ability to apply our emulation strategies in neuroscience, using electrical features generated by the NEURON simulator for Neocortical L2/3 Large Basket Cells. The first generation included 1,000 individuals and 28 output features. Nineteen features were observed and recorded for all the individuals. For nine features, though, all from the IDRest seven current group, the event that defines the feature did not occur in some of the individuals, hence no value could be recorded; for example, if an individual simulated only one spike, the minimal voltage between spikes is undefined. We refer to these cases as missing values (see Table 1) and propose an emulation strategy for them in the section on Emulation of Features with Missing Values.

**Table 1:** Features with missing values (generation 1).

**Feature name**	**Number of missing values**
Peak voltage	11
AP amplitude	874
AHP depth (absolute)	11
Burst number	933
ISI log slope skip	895
Spike half width	11
Minimal voltage between spikes	737
AP2 AP1 peak difference	737
Time to second spike	737

There is strong correlation between features of the same type, but from different current groups (see Supplementary Figure 1). Thus, it is sufficient to illustrate the potential of emulation on a single current group; we used the subthreshold current injection of 0.04 nA for 1,000 ms (IVF_3).

Two of the features, the number of initial spikes and the spikecount, have distributions concentrated near 0, with spikes present in only a few individuals. For most stimulus protocols, the laboratory target values for these features are 0 (see Appendix B), so the optimization process encourages these outcomes and zero values are prevalent in all generations. In the IDRest_7 stimulus protocol, these features have positive laboratory target values (6 and 55.6, respectively), so later GA generations have more individuals with positive values.

**Figure 4:**
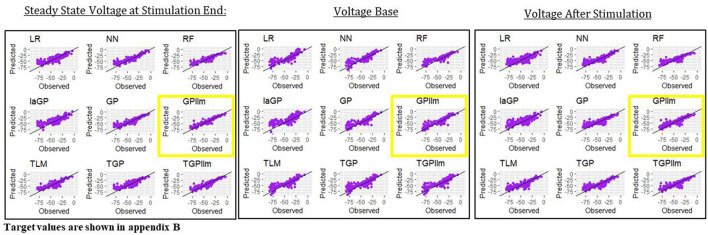
Observed (i.e., simulated) vs. predicted (i.e., emulated) values for the three electrical features for each of the nine emulation methods considered here.

This section presents emulators that use 80% of the individuals in a generation (here 800 individuals) as the train set, with 20% set aside as a test set. Figure 4 and Table 2 present results for three representative features (steady state voltage at the end of the stimulation, voltage base, and voltage after stimulation) and for each of the nine emulators that were described in the methods section. Table 3 presents the standard deviation of the simulated outcomes for each feature and thus serves as a benchmark for the root mean squared errors of the emulators in Table 2.

**Table 2:** RMSE and run time for emulating generation 1 features.

**Model**	**Steady state voltage**		**Voltage base**		**Voltage after**	
	**at stimulation end**				**stimulation**	
	**RMSE**	**Time (Min)**	**RMSE**	**Time (Min)**	**RMSE**	**Time (Min)**
LR	9.45	0.00	10.64	0.00	10.95	0.00
NN	7.67	0.44	9.37	0.47	8.72	0.44
RF	7.40	0.06	8.64	0.06	8.55	0.06
laGP	11.10	0.81	12.89	0.58	11.83	0.79
GP	7.63	4.04	9.22	4.82	8.90	3.42
GPllm	**6.11**	**3.77**	**8.19**	**3.93**	**8.10**	**3.01**
TLM	8.07	0.07	10.05	0.06	9.08	0.06
TGP	8.88	3.20	10.10	4.86	8.85	2.07
TGPllm	6.80	4.05	10.08	3.84	8.37	3.32

*For each feature, the results for the method with the most accurate predictions are in bold font*.

**Table 3:** Standard deviations for the 3 features in generation 1.

**Steady state voltage at stimulation end**	**Voltage base**	**Voltage after stimulation**
15.94	17.88	16.75

The most accurate emulator for all three features is GPllm, as seen in Figure 4 and Table 2. The RMSE of this emulator is less than one-half the SD for each feature, indicating that it explains at least 75% of the variation in the independent test data. The fastest run times were for LR, NN and RF, <1 min on all features. The GP/TGP models average 1–4 min, still very fast when compared to the run time of the simulator.

### Emulation of Features With Missing Values

**Figure 5:**
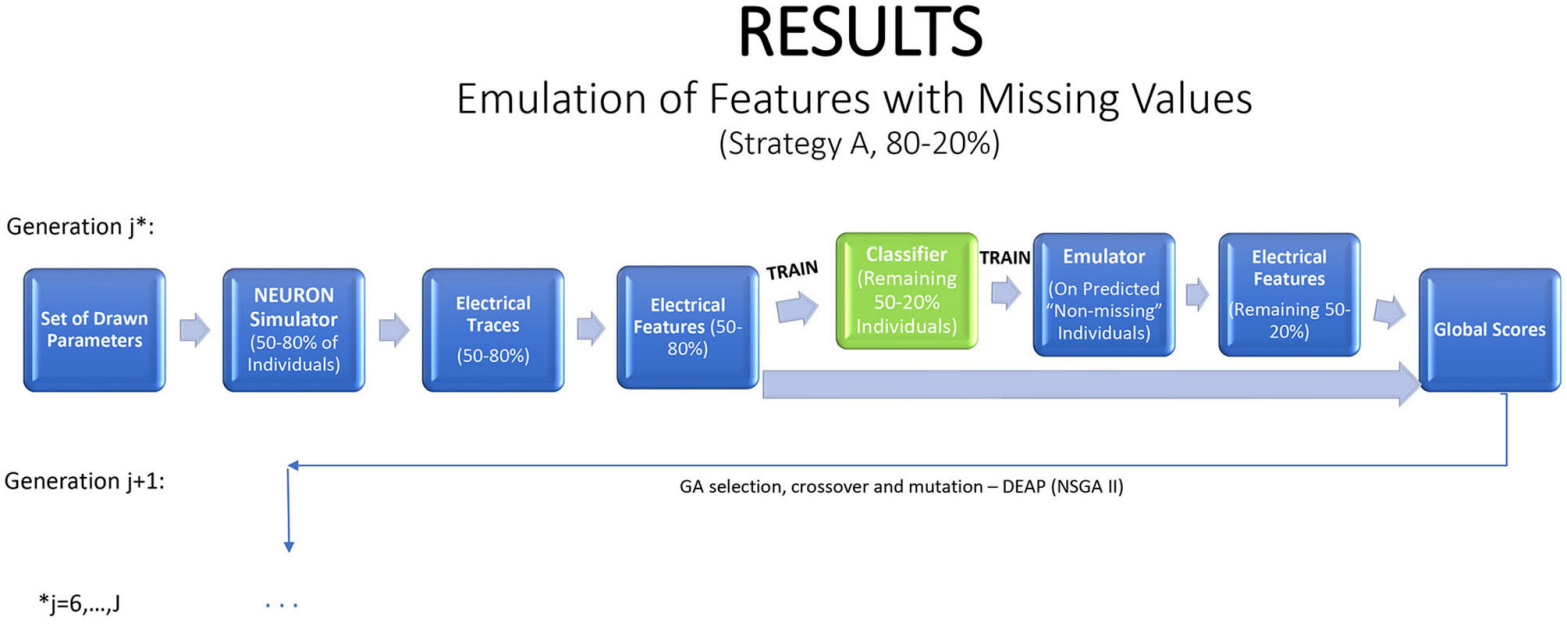
A strategy for a modified genetic algorithm for features that are observed only in some of the traces. The strategy simulates traces for 80% of the individuals, using them to fit a classifier, which is applied to the remaining 20% of the individuals. If the classifier predicts that the feature will be observed, it is then predicted with an emulator.

Features with missing values present an interesting problem. An ideal emulator for a feature with some missing values would predict whether or not it is missing, and what is its value if it is not missing. Standard emulator approaches are not geared to this task. Here we propose and examine a two-stage emulation strategy. First, we fit a classifier that predicts whether an individual is likely to produce a missing value when simulated in NEURON. Second, we fit an emulator only to those individuals that supplied data. The emulator cannot be expected to give satisfactory results outside the input region where training data were obtained. We minimize this risk of extrapolation by predicting results only for new individuals classified as non-missing. The resulting combined emulator requires a classifier that is sensitive (in which it predicts an observed value for most individuals that actually produce a value in NEURON), and an accurate emulator when data are present. An illustration of this method is provided in Figure 5.

We apply the method to three features from IDRest 7 that contain missing values: peak voltage, AP amplitude, and minimum voltage between spikes. The chosen starting point for the application is generation 6, due to a sharp decrease of missing values in this generation.

A standard RF classifier was trained and applied after briefly tuning the number of trees (*N*_*tree*_) and number of features per tree (*mtry*) hyperparameters in generation 6 on AP amplitude and minimum voltage between spikes. The optimal solutions were *N*_*trees*_ = 1, 200, *mtry* = 15 on AP amplitude and *N*_*trees*_ = 1, 100, *mtry* = 24 on minimum voltage between spikes. Note that no classifier was applied to peak voltage since it had only five missing values in this generation. The results on the 200 independent test individuals are shown in Tables 4, 5.

**Table 4:** RF classifier confusion matrix for AP amplitude in generation 6.

**AP Amplitude**		**Observed**	
		**Non-missing**	**Missing**
Predicted	Non-missing	166	1
	Missing	6	27

**Table 5:** RF classifier confusion matrix for minimum voltage between spikes in generation 6.

**Min voltage between spikes**		**Observed**	
		**Non-missing**	**Missing**
Predicted	Non-missing	123	24
	Missing	7	46

The accuracy of the AP amplitude classifier is 96.5%, and the accuracy of the minimum voltage between spikes classifier is 84.5%. In this use case, as noted earlier, the most important property of the classifier is high sensitivity (i.e., that individuals with values are correctly classified as non-missing). Erroneously classifying them as missing would discard individuals that produce a valid, maybe promising, value, without running them through the emulator. The sensitivity of the AP amplitude classifier on the test data is 96.5%, and the sensitivity of the minimum voltage between spikes classifier is 94.6%. These are very good results, illustrating the ability of the RF classifier to predict whether or not individual outcomes will be missing. For both features, the classifiers pass on to the emulator most of the valid individuals. The specificities of the two classifiers are 96.4 and 65.7%, respectively. The classifier for AP amplitude passes on only a small fraction of those with missing values, but the minimum voltage classifier errs on about one-third of the individuals that are missing the outcome. This error is less serious in the context of MOO, hence still reasonable for applications.

**Figure 6:**
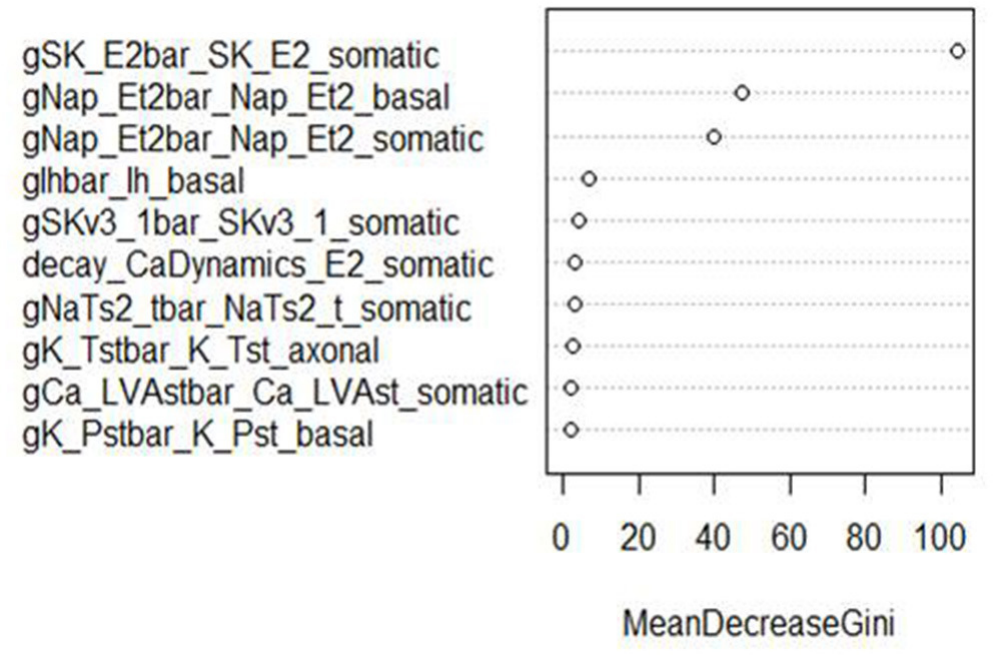
Variable importance plot for the RF classifier of AP amplitude, using the data from generation 6.

**Figure 7:**
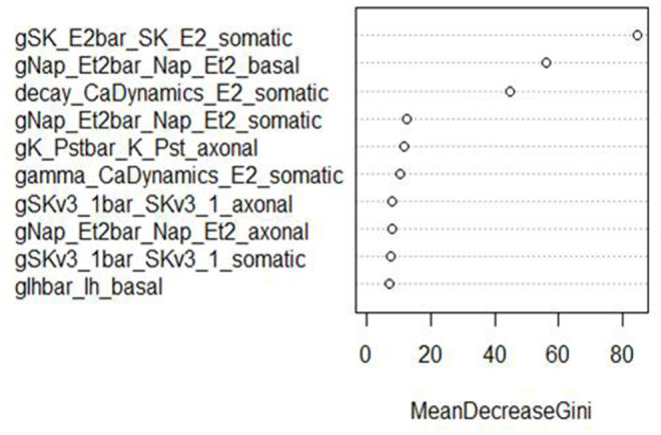
Variable importance plot for the RF classifier of minimum voltage between spikes, using the data from generation 6.

In addition, RF classifiers measure variable importance by the average decrease in Gini impurity over all splits (Menze et al., 2009). Figures 6, 7 show the 10 most important parameters for both RF classifiers.

The variable importance lists could be used to explore the parameter space more efficiently. The variable importance plot presented in Figure 6 shows that “gSK_E2bar_SK_E2” from the “somatic” parameter group is the most influential for AP amplitude, followed by “gNap_Et2bar_Nap__Et2” from the “basal” and “somatic” parameter groups. Interestingly, the variable importance list for the second feature, minimum voltage between spikes, is very similar to the first list. Although the same parameters dominate both outcomes, these features are not typically missing (or present) in the same individuals; they have the same status on 514 training individuals, but differ on 486. Each is missing much more often when the other is present than when it is missing.

The top two explanatory variables for these features are the same, suggesting that they are very influential on spike formation/pattern in general. These insights can be used to guide the GA algorithm search to areas with fewer missing values, enhancing efficiency by increasing the chance of finding promising individuals.

Following the method set out in Figure 5, an emulator can now be applied to the non-missing predicted individuals for each feature. In this proof-of-concept application, the emulator was trained and applied to all non-missing simulated individuals. This section shows the results of our emulation strategy on these non-missing individuals in generation 6 for the three selected features.

**Figure 8:**
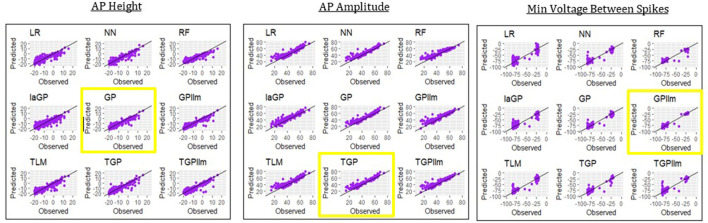
Observed (i.e., simulated) vs. predicted (i.e., emulated) values for the three electrical features which are not always observed, using the data from generation 6, for each of the nine emulation methods considered here.

Table 6 and Figure 8 show that the most accurate emulator for peak voltage is GP, for AP amplitude it is TGP, and for minimum voltage between spikes it is GPllm. Figure 8 shows a good fit of all these emulators to the relevant electrical features. The RMSEs of the emulators are less than half as large as the standard deviations of the features (Table 7), indicating ability to achieve accurate emulation. Second, Table 7 shows that the run times of the LR, NN, and RF models for these features are very fast. The GP/TGP models are slower for this task, with average run times of 1–14 min, but that is still substantially faster than the run time of the simulator.

**Table 6:** RMSE and run time for emulating generation 6 features.

**Model**	**Peak voltage**		**AP amplitude**		**Min voltage**	
					**between spikes**	
	**RMSE**	**Time (Min)**	**RMSE**	**Time (Min)**	**RMSE**	**Time (Min)**
LR	4.9	0.0	6.95	0.00	11.77	0.00
NN	3.72	0.74	6.24	0.35	8.01	0.38
RF	3.95	0.08	6.13	0.05	6.31	0.05
laGP	4.57	0.73	6.71	0.55	10.76	0.44
GP	**3.46**	**13.73**	6.07	2.03	6.99	2.05
GPllm	3.47	13.08	6.47	1.72	**4.89**	**1.61**
TLM	4.21	0.12	6.95	0.03	9.69	0.05
TGP	3.68	5.14	**6.01**	**3.63**	11.60	1.01
TGPllm	3.74	11.76	6.33	4.61	9.25	2.14

*Target values for each feature are shown in Appendix B. For each feature, the results for the method with the most accurate predictions are in bold font*.

**Table 7:** Standard deviations table (#2).

**Peak voltage**	**AP amplitude**	**Min voltage between spikes**
8.57	13.31	26.7

Table 6 and Figure 8 show that the most accurate model for minimum voltage between spikes was GPllm, yielding the lowest RMSE for this feature-−4.67. Surprisingly, this model is far more successful than any of the other emulators for this electrical feature, unlike the other features, in which the competition between all models is relatively close. This is surprising given the bi-modal distribution of this feature, which makes it a challenging task and led us to expect that only tree-based emulators would successfully capture the two distinct output regions (near −80 and −25). However, GP and GPllm proved that the flexibility of the GP models, discussed in chapter 2, can overcome these challenging gaps and fit the data well.

### Inference

One advantage of GP emulators is that they provide full predictive distributions, and not just point predictions as with RF or NN models. The distributions provide a basis for uncertainty assessments and Bayesian sensitivity analysis. We demonstrate the ideas here for selected features, using the most accurate GP model for each feature.

#### Predictive Uncertainty

**Figure 9:**
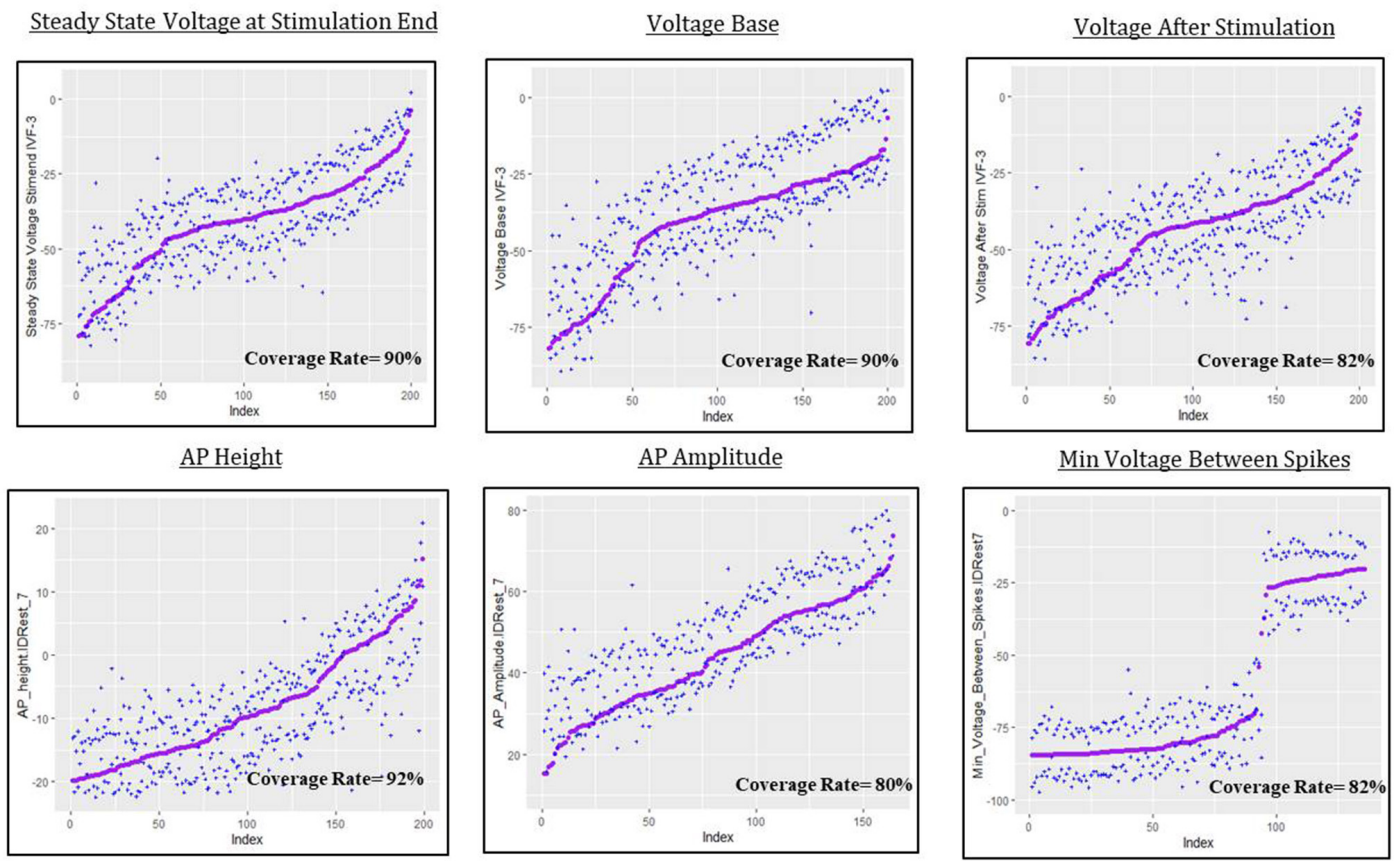
Predictive uncertainty plots for GP emulator predictions of the six electrical features considered here.

The predictive uncertainty assessment in this section is given by plots like Figure 9 that take into account the real simulated responses (purple points) and the estimated 0.05 and 0.95 quantiles, ${\hat{q}}_{5\%}\;$ and ${\hat{q}}_{95\%},$ (blue crosses). Coverage describes the fraction of test individuals whose outcomes are within the estimated quantiles. The nominal coverage rate here is 90%, so coverage at that level indicates both a good fit of the model to the data and an accurate assessment of model uncertainty.

For steady state voltage at stimulation end, voltage base and peak voltage the coverage rate is very close to 90%. For the other features, the coverage rate is closer to 80%, which indicates that the intervals are a bit too narrow or, as for AP amplitude, that they suffer from bias, moving the quantiles off target (in this case slightly “high”). The most interesting result in this section is the coverage plot of minimum voltage between spikes. The GPllm model, in addition to emulating the two distinct components of the simulated responses, also provides a realistic picture of the uncertainty, and clear discrimination between settings with results near the higher/lower mode.

The spreads of these interval widths for each feature were relatively small—all intervals were about 1–2 standard deviations in width, with respect to the corresponding standard deviations for each feature (shown in Tables 3, 7). Thus the statistical model's predictive intervals (e.g., predictive uncertainty measurements) yield good coverage while remaining narrow.

#### Bayesian Sensitivity

Sensitivity analysis provides a summary of the importance of each feature in determining the output. We add to the actual input factors two noise factors (random samples from a standard normal distribution); these factors act as “negative controls” in the plots, providing a frame of reference for assessing the actual factors. The main effects plots of selected parameters from these Bayesian sensitivity analyses are in the Supplementary Material (Supplementary Figures 2–5).

**Figure 10:**
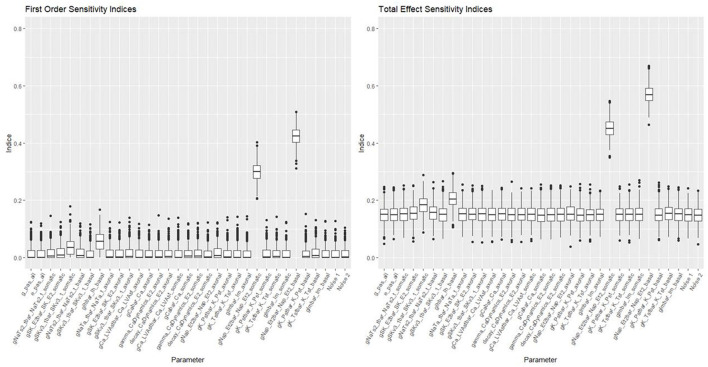
Sensitivity indices for each input factor in emulating AP amplitude. The panel on the left shows first-order indices, and the panel on the right shows total effect indices. The last two factors on the right are randomly added “noise factors,” which indicate what might be expected for a factor that, by design, has no impact at all on the feature.

**Figure 11:**
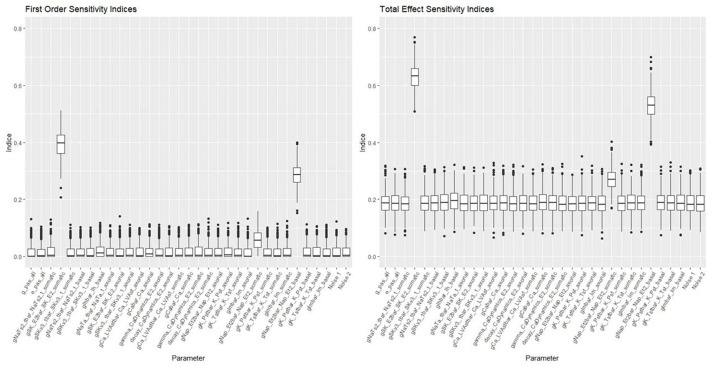
Sensitivity indices for each input factor in emulating minimum voltage between spikes. The panel on the left shows first-order indices, and the panel on the right show total effect indices. The last two factors on the right are randomly added “noise factors,” which indicate what might be expected for a factor that, by design, has no impact at all on the feature.

Figures 10, 11 show the first order and total sensitivity indices of all 31 parameters for AP Amplitude and Min Voltage Between Spikes. These indices were calculated using the *sens* function in the tgp package using 80% of generation 6 as the training data and the most accurate models (TGP model for AP amplitude and a GPllm model for minimum voltage between spikes). The most prominent parameters were gNap_Et2bar_Nap_Et2_somatic and again gNap_Et2bar_Nap_Et2_basal for AP amplitude, and gSK_E2bar_SK_E2_somatic and gNap_Et2bar_Nap_Et2_basal for minimum voltage between spikes. The second most influential parameter for each feature was one of the two most influential parameters on the other feature—gSK_E2bar_SK_E2_somatic for minimum voltage between spikes, and gSK_E2bar_SK_E2_somatic (along with gSKv3_1bar_SKv3_1_basal) for AP amplitude. The presence of common factors is surprising, as the Pearson correlation coefficient between these two outputs is only −0.55.

These parameters were also the most influential ones for the RF classifier (presented in Figures 7, 8) of presence/absence. For AP amplitude, the most influential RF parameter is gSK_E2bar_SK_E2_somatic (the fourth most influential parameter according to the sensitivity analysis) followed by gNAP_Et2bar_Nap_Et2_basal and gNAP_Et2bar_Nap_Et2_somatic (the two leading parameters in the sensitivity analysis). For minimum voltage between spikes, the most influential RF parameters are gSK_E2bar_SK_E2_somatic (the most influential parameter in the sensitivity analysis), followed by gNAP_Et2bar_Nap_Et2_basal (the second most influential parameter in the sensitivity analysis) and decay_CaDynamics_E2_somatic (not influential in the sensitivity analysis).

These results offer interesting insights on the electrical features studied here. First, the correlation between the influential parameters on the classification and the regression tasks implies that there is a connection between the numeric result of the feature and its tendency to result in missing/non-missing output. In addition, the similarity between the influential parameters for AP amplitude and minimum voltage between spikes implies that this set of parameters (gSK_E2bar_SK_E2_somatic, gNap_Et2bar_Nap_Et2_somatic, gNAP_Et2bar_Nap_Et2_basal and maybe also gSKv3_1bar_SKv3_1_basal and decay_CaDynamics_E2_somatic) may be very influential for other features, so they should be explored carefully throughout the optimization process.

## Discussion

The paper presents strategies for using statistical emulators as a tool in studying neural processes *via* simulation platforms. Our results show that statistical emulators can effectively mimic the results from a complex neural simulator with many input factors and show promise for use as an integral tool within the framework of MOO.

The most popular emulation approach in the statistical literature is the GP model. Our results show that GP models, especially when modified as TGP (Gramacy and Lee, 2008) and with/without limiting linear models, can indeed provide excellent results for modeling the electrical features generated by the NEURON simulation platform for neocortical L2/3 large basket cells. Since each generation contained no more than 1,000 valid (non-missing) observations, the run times of these models make them practical choices for use as emulators. They required 1–15 min run time, even though applied on far simpler and weaker computers than those available within the HBP community and to most neuroscience research teams. The overall performance, in terms of accuracy, adaptivity and efficiency of these models was remarkable and can be a valuable tool when implemented in similar future neuroscience applications.

It was challenging to fit a good emulator to features with only a small spread of feature values from the simulator (and where the laboratory target values are different from 0). In these cases it is recommended to wait until more spread is observed, and only then to fit an emulator. This suggestion holds also for features observed in the laboratory that have many missing values at the start of the optimization process (as shown in Table 1; Supplementary Figure 1), but quickly advance to individuals with far fewer missing values.

Another important aspect of the sample size is the train-test ratio. For our use case, we chose an 80–20% ratio. Smaller ratios are of course possible and will accelerate the computation, but could degrade accuracy. The best choice will typically depend on the application and should be made to efficiently balance the time-accuracy tradeoff. It may depend on the goal of the emulation.

There are several important advantages of the GP model. It offers a flexible solution for dependent observations with numerous kernel options that can fit functions of various degrees of smoothness. In addition, unlike current black-box models, the GP model is easily interpretable and offers not only point predictions, but also uncertainty assessment and inference for the simulator values at a given input location.

The basic GP model requires adjustments to handle training data with a large number of individuals. Several useful methods have been proposed that make it possible to fit GP models to training data with tens and even hundreds of thousands of observations. These include the multi-step approach (Haaland and Qian, 2011) and the sub-design and local models approach (Gramacy and Apley, 2014; Park and Apley, 2018). Some of these methods were applied to the data (using the laGP package) in this paper, but were not among the better-fitting emulators. Further research is needed to elucidate the accuracy of these emulation methods.

Our research illustrates the power of emulators based on data that came from GA iterations to identify individuals with good fit to observed data. In this accompanying role, emulators can be catalysts to the optimization process. Emulator use can go much further, guiding the process toward more promising regions of the parameter space. The emulator can serve as a filter to identify promising individuals at the beginning of the next generation. This amounts to a role reversal between the emulator and the simulator–first the emulator filters individuals, then the simulator kicks in. Pre-screening and selection of individuals by the emulator may lead to over-optimistic bias. Techniques could be developed to adjust for these biases. In addition, the drift toward better individuals will lead to new regions of the parameter space where the emulator may no longer be accurate. Strategies will be needed for when to abandon an emulator and to train a new one using more recent data.

Second, the inference products that accompany GP models can be used to guide a genetic algorithm toward more promising areas in the parameter space. The uncertainty of each prediction can be accounted for, alongside its closeness to the laboratory target value, when the next generation's parents and individuals are selected.

Third, fitting an emulator provides valuable information on the relationship of each input parameter to the output. Knowing which parameters are most influential for each feature can help focus attention on these parameters when forming the next generation. As seen in our sensitivity analysis and in our factor influence summaries, often just a few of the parameters have large effects.

Our emulators, like MOO, focus on features derived from the electrical traces produced by NEURON, i.e., low-dimensional multivariate output. We chose to model each outcome feature separately. The correlations observed among the features in the training data were used only to reduce the dimension, choosing representatives of groups with high correlation. Alternatively, we could have directly applied methods for multivariate emulation (see for example, Rougier, 2008; Overstall and Woods, 2016).

There is a substantial body of research on methods for analyzing and emulating functional data (Ramsay, 2005; Bayarri et al., 2007, 2009; Higdon et al., 2008; Levy, 2008; Goh et al., 2013; Hung et al., 2015; Plumlee et al., 2016; Chakraborty et al., 2017; Salter et al., 2019). In principle, these methods could be used to directly emulate the electrical traces. However, all these methods assume that the output is a smooth function. That assumption is clearly inappropriate for the electrical traces obtained here, but might be perfectly acceptable for other types of functional output in neural simulations.

## Conclusions

This paper provides a first and crucial step illustrating the potential value of fitting statistical emulators to data arising from complex neuroscience simulation engines. The test case shows that accurate emulation is possible and propose strategies for incorporating emulation within optimization processes. We proposed different statistical emulation strategies, addressing general challenges of computer models such as: where to sample the input space for the simulator, how to make sense of the data that is generated, how to estimate unknown parameters in the model and how to model features that do not always occur in a simulation.

In particular, we derive accurate emulators from modest training samples of features generated by the NEURON simulator for neocortical L2/3 large basket cells. We compared different statistical emulation methods in terms of both accuracy and efficiency (i.e., run times). Our main conclusion is that statistical emulators, mainly GP models, can accurately mimic the simulator's outputs. Thus emulation can expedite processes that require intense use of the simulators (such as MOO iterations). Emulators can dramatically reduce computation time and so accelerate these processes.

The methods we present showed great value in two aspects. First, they demonstrated the ability to accurately predict the simulator's outcome for different electrical features at different stages (i.e., generations), thus offering a novel method to accelerate the optimization process of simulators, especially when these require many evaluations. Second, they are able to identify which inputs are the most influential ones, thanks to the chosen statistical models (variations of Gaussian Process Models) which support this kind of inference. The first aspect helps to address the computational issues inherent in the speed challenge of Makin (2019); the second aspect is relevant both to the scale and complexity challenges described there.

This research presents useful first steps toward exploiting emulators in neuroscience research. There are numerous directions that could fruitfully be taken in future work. One important area for neuroscience is to adapt the models for use with features that have many missing values, when a two-phase emulation (classification—regression) is required, and on other statistical models that can improve the performance of the presented emulators. Another is to consider multivariate or functional simulator output. An important step ahead will be to adapt and improve optimization methods that fully and automatically incorporate emulation for application to neuroscience research.

## Data Availability Statement

Our work is on a very limited subset of data generated for a previous paper. Requests to access these datasets should be directed to GS, giladshapira@mail.tau.ac.il.

## Author Contributions

All authors listed have made a substantial, direct, and intellectual contribution to the work and approved it for publication.

## Funding

This research was supported by funding from the European Union's Horizon 2020 Framework Programme under the Specific Grant Agreements Nos. 785907 and 945539 (Human Brain Project) and, to IS, a grant support from the Drahi family foundation, the Gatsby Charitable Foundation, and by funding to the Blue Brain Project, a research center of the École Polytechnique Fédérale de Lausanne (EPFL), from the Swiss government's ETH Board of the Swiss Federal Institutes of Technology.

## Conflict of Interest

The authors declare that the research was conducted in the absence of any commercial or financial relationships that could be construed as a potential conflict of interest.

## Publisher's Note

All claims expressed in this article are solely those of the authors and do not necessarily represent those of their affiliated organizations, or those of the publisher, the editors and the reviewers. Any product that may be evaluated in this article, or claim that may be made by its manufacturer, is not guaranteed or endorsed by the publisher.
