# Supplementary material for: Statistical Emulation of Neural Simulators: Application to Neocortical L2/3 Large Basket Cells
Source: Front Big Data. 2022 Mar 25;5:789962. doi: 10.3389/fdata.2022.789962 (PMC8992430; doi:10.3389/fdata.2022.789962)
Supplement: Supplementary file 1 [file Data_Sheet_1.pdf]

1. Methodological development of sensitivity indices

The following brief description of the Saltelli approach to sensitivity analysis closely follows Gramacy (2020) (chapter 8.2), which can be read for more details. This method of sensitivity analysis is based on a reference distribution for each input variable X_j, sometimes called an uncertainty distribution $U_j(x_j)$. U can represent uncertainty about future values of x, or the relative amount of research interest in various areas of the input space. In many applications, the uncertainty distribution is simply uniform over a bounded region. When experimental data have supplied information about *X*, *U* might be the resulting posterior distribution. First, it will be assumed, temporarily and for computational simplicity, that all inputs are independent, so that:

$$\boldsymbol{U(x) = \prod_{k=1}^{m} u_k(x_k)}, \tag{S1}$$

where u_k for $k = 1, \dots, m$ represent densities assigned to the margins of x. The simplest sensitivity indices are ***main effects***, which deterministically vary one input variable while averaging over all others. Then, the main effect function of X_j for the output *Y* can be written as:

$$\boldsymbol{me(x_j) = E_{U_{-j}}\{y|X_j = x_j\} = \int\int yp(y|x_1, \dots, x_m)dy \prod_{k\neq j} u_k(x_k)\, dx_{-j}}. \tag{S2}$$

The notation $E_{U_{-j}}\{y|X_j = x_j\}$ represents the expectation of y conditional on $X_j = x_j$, averaging over all other variables. If $y(x)$ is a deterministic realization of the simulator, the term $p(y|x_1, \dots, x_m)$ and the integration over y in Eq. S2 are replaced by the fixed value $y(x_1, \dots, x_m)$.

Computation of main effects, and other terms in sensitivity analysis, can often be accelerated by replacing y in Eq. S2 by an emulator, $\hat{y}(x)$. The emulator might be a random variable (for example from a GP emulator) and not a deterministic function of the input. In that case the term $p(\hat{y}|x_1, \dots, x_m) = p(\hat{Y}|x) = p(\hat{Y}(x) = \hat{y})$ and the integration over $\hat{y}$ will generate the conditional expectation of y given $X_1 = x_1, \dots, X_m = x_m$. When using a surrogate to generate $\hat{y}(x)$, such as a GP predictor, one can sometimes obtain a distribution of $\hat{y}(x)$ from the surrogate (unless the surrogate only produces a point prediction). Thus, if the simulator is expensive to run, one can approximate the main effect using a GP surrogate, and even though it requires averaging over the posterior of y, it may offer a much easier and cheaper method to compute the main effects. The advantages and drawbacks of using an emulator to approximate the main effect, instead of computing it from the actual simulator values, will be further discussed later in this chapter.

The averaging here is both on all possible values of y and all possible values of $x_1, x_2, \dots, x_{j-1}, x_{j+1} \dots x_m$. Note that even though the domains of the input variables are continuous, it is often not possible to compute these integrals analytically, so in practice it is common to use quadrature formulas or Monte Carlo that rely on evaluations of these variables over a grid of points, and then averaging by

summation and not integration. In addition, even though Eq. S2 (Eq. 8.5 in Gramacy, 2020) refers to the mean main effects ($\mu(x) = \int yp(y|x)dy$), any aspect of $p(y|x)$ that can be expressed as an integral can be averaged with respect to U. In particular, Gramacy (2020) computes the predictive 0.05 and 0.95 quantiles, an idea that is applied here, as well, with the notation $\hat{q}_{5\%}$and $\hat{q}_{95\%}$ for the two quantiles.

It is important to notice that me is a function, and not the sensitivity index. For each input variable, this function can be plotted for different values of the input variable to provide a graphical illustration of its impact on the output variable y. Large variations of the image of the me function for different locations of the input variable indicate that the input variable is very influential on y (since it affects the conditional expectation of y). The form of the effect of each input variable can also be learned from the form of the me function plots- which may point to a linear effect, a sinusoidal effect, or some other form of dependence. In contrast, these plots can point out that a certain input variable has little or no influence on the outcome variable, when the me function plot seems pretty much flat in different locations of the input variable. In addition, when the main effect for the predictive $\hat{q}_{5\%}$ and $\hat{q}_{95\%}$for the variable are close to the main effects for the predictive mean, it indicates that the predictive uncertainty of y, once X_j is fixed on some specific value x_j, is small, so it is mostly explained by this input variable.

The above approach can be extended from main effects to interactions. The most common notion of sensitivity indices is tied to the relationship between conditional and marginal variance for y. This variance-based approach is derived by decomposing , a deterministic objective function into summands of functions on lower dimensional subsets of the input space (Sobol (1993, 2001)). Consider the functional decomposition:

$$\boldsymbol{f(x_1, \dots, x_m) = f_0 + \sum_{j=1}^{m} f_j(x_j) + \sum_{1 \le i < j \le m} f_{ij}(x_j, x_i) + \dots + f_{1,\dots,m}(x_1, \dots, x_m)}. \quad \text{(S3)}$$

The functions are defined in terms of expectations of f, over subsets of the inputs with respect to the distributions $U(x)$. Note that the second component of eq. (S3), $\sum_{j=1}^{m} f_j(x_j)$, is the sum of the me functions defined above. Sobol-based sensitivity analysis attempts to decompose, and quantify, variability in $E\{y \mid x_J\}$ with respect to changes in x_J according to $U_J(x_J)$. Note that x_J is now a subset of covariates $x_J = \{x_j : j \in J\}$ where $J \subseteq \{1, \dots, m\}$. If U is such that inputs are independent, the variance decomposition is available as:

$$\boldsymbol{Var\left(E_{U_{-J}}\{y|x\}\right) = \sum_{j=1}^{m} V_j + \sum_{1 \le i \le j \le m} V_{ij} + \dots + V_{1,\dots,m}}, \quad \text{(S4)}$$

where $V_j = Var_{U_j}\left(E_{U_{-j}}\{y|X_j = x_j\}\right)$, $V_{ij} = Var_{Ui_j}\left(E_{U_{-ij}}\{y|X_i = x_i, X_j = x_j\}\right) - V_i - V_j$ and so on. This is very similar to the known ANOVA decomposition of the total sum of squares in the response variable. When inputs are correlated this

identity no longer holds, although a "less-than-or-equal-to" inequality is always true. Nevertheless, it's useful to retain an intuitive interpretation of the V_J's as a portion of overall marginal variance. With that motivation in mind, the first-order sensitivity indices are defined as:

$$\boldsymbol{S_j = \frac{V_j}{Var_U(f)}, j = 1, \dots, m}. \quad (\text{S5}).$$

S_j is the proportion of variability in the response attributable to the jth input, i.e., response sensitivity to variable main effects. S_j is a scalar, not a function, so a first-order analysis reports m numbers, which is of lower dimension than main effects, providing m functions. The total sensitivity indices are defined as:

$$\boldsymbol{T_j = \frac{E\left\{Var_{U_{-j}}(y|X_{-j}=x_{-j})\right\}}{Var_U(y)}}, \quad (\text{S6})$$

where $(X_{-j} = x_{-j})$ means $(X_1 = x_1, X_2 = x_2, \dots X_{j-1} = x_{j-1}, X_{j+1} = x_{j+1}, \dots X_m = x_m)$.

Observe that:

$$\boldsymbol{E\left\{Var_{U_{-j}}(y|X_{-j} = x_{-j})\right\} = Var_U(y) - Var_U(E_U(y|X_{-j} = x_{-j})}. \quad (\text{S7})$$

According to the law of total variance, $\boldsymbol{T_j}$ measures residual variance in conditional expectation and thus represents all influence connected to a given variable. Consequently, the difference between first-order and total sensitivities, $\boldsymbol{T_j - S_j}$, measures variability in $\boldsymbol{y}$ due to the interaction between the j'th input and the other inputs. A large difference $\boldsymbol{T_j - S_j}$, can trigger additional local analysis to determine its functional and interactional form. Again, note that if the simulator is expensive to run, one can approximate these indices using the distribution of the surrogate, i.e. the GP predictor.

The **tgp** package in R was used to implement sensitivity analysis in this research.

2. Proposed priors and posterior estimation techniques for Bayesian Treed Models

We present here the prior proposed by Chipman et al., (2002), along with their methods for carrying out the analysis with it.[1] The proposed prior *p(T)* is implicitly defined by a tree-generating stochastic process that "grows" trees from a single root tree by randomly "splitting" terminal nodes. A tree's propensity to grow under this process is controlled by a two-parameter node splitting probability -

$$\boldsymbol{p(node\ splits|depth = d) = \alpha(1 + d)^{-\beta}} \qquad \text{(S8)}$$

The parameter α is a "base" probability of growing a tree by splitting a current node and β determines the rate at which the propensity to split diminishes as the tree gets larger. The common choice of α and β, verified by Gramacy & Lee (2008) for their model, is 0.5 and 2, respectively. At each node, the variable and the cut-off value are chosen from a uniform distribution.

For $p(\boldsymbol{\Theta} \mid \mathrm{T})$, the model space is too large to obtain the posterior information directly. Imagine a shallow tree formation, with only one split, and a dataset with only 2 features, each with 10 levels. These settings allow for the creation of 20 different trees. In large datasets, there are more than 2 features, and more than 10 levels (especially when features are continuous). Thus, the number of possible trees, although finite, is extremely large, and the posterior information cannot be obtained directly.

Chipman et al., (2002) proposed a Markov Chain Monte Carlo based method that stochastically searches for high posterior trees *T*. The first step of their method integrates $\boldsymbol{\theta}$ out of the posterior so only T remains. Then the conditional marginal distribution of T given the observed data is used as a limiting distribution for a Metropolis-Hastings search algorithm, which simulates a Markov chain T^0, T^1, T^2 and so on. The search starts with an initial tree T^0. Then a new candidate tree is generated by randomly growing/pruning/changing the splitting rule of one terminal node of T^0, or randomly swapping two nodes of T^0. This candidate is assigned with a transition probability that accounts for the posterior density (evaluated as the product of the tree's likelihood and its prior density) of T^0 and the new candidate. Thus, the chain simulated by this algorithm should move quickly towards a region where $p(T|\boldsymbol{X}, \boldsymbol{y})$ is large, and at the same time avoid lingering in regions with local maxima.

Chipman et al., (2002) noticed that even though this search algorithm does tend to quickly gravitate towards a region where the posterior $P(T|\boldsymbol{X}, \boldsymbol{y})$ is large, it tends to move locally in that region for a long time and does not visit other peaks of this multimodal posterior. Thus, they recommend starting the search with multiple restarts of the algorithm, saving the most promising trees from each run. Finally, Chipman et al., (2002) chose to fit a hierarchical linear regression to each terminal node of these most promising trees. They demonstrated excellent prediction accuracy on three

[1] https://doi.org/10.1023/A:1013916107446

different examples compared to other statistical models, including a conventional tree model in which the prediction in each terminal node is a constant.

3. Development of TGP models

Given a tree, T, that splits the input space into R non-overlapping regions $\{r_v\}_{v=1...R}$, a fully Bayesian hierarchical generative GP model, which includes a stationary process $\boldsymbol{f}$ and a linear regression product, is fitted to each region. The fully Bayesian hierarchical generative GP model assigns a prior distribution to each of the hyperparameters of $\boldsymbol{f}_v$, where the correlation matrix K_v is taken to be from the separable power family with a known power but unknown length scale and nugget hyperparameters. Gramacy & Lee (2008) proposed to assign a mixture of Gammas priors for the length-scales and an exponential prior for the nugget.

In addition, normal prior distributions are also assigned to each of the linear regression coefficients - $\boldsymbol{\beta}_v \sim \boldsymbol{N}(\boldsymbol{\beta}_0, \sigma_v^2 \tau_v^2 \boldsymbol{W})$**,** and each of the hyperparameters of this distribution is also assigned a common prior distribution. The hyperparameters of these prior distributions are treated as known and form the upper level of the hierarchy.

Samples from the joint posterior distribution of $\boldsymbol{\theta}, T$ are gathered by alternately drawing $\boldsymbol{\theta}|T$ and $T|\boldsymbol{\theta}$. The tree formation process is very similar to the grow/prune/change/swap MCMC algorithm of Chipman et al., (2002), with small changes. After the formation of a tree, the data from each of its R regions $(r_{1..v})$ is used to update the GP parameters and hyperparameters. Samples from the posterior distribution of $\boldsymbol{\theta}|T$ are gathered using MCMC by conditioning on the upper level hyperparameters and drawing θ_v, and then drawing the upper level hyperparameters. All parameters can be sampled with Gibbs steps, fully detailed in Gramacy (2005) and Gramacy & Lee (2008), except those that parameterize the covariance function K, e.g. the length scales and nugget hyperparameters, which require Metropolis-Hastings (MH) draws.

4. Correlation Between Features:

Figure S1- Features histograms and correlations (by type)

Number of initial spikes:

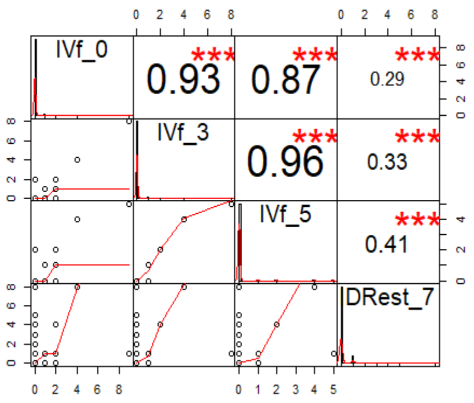

Steady state voltage at stimulation end:

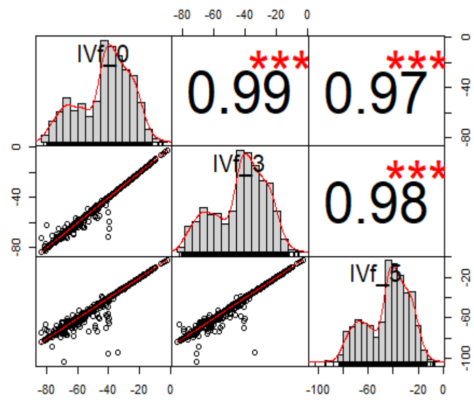

Voltage base:

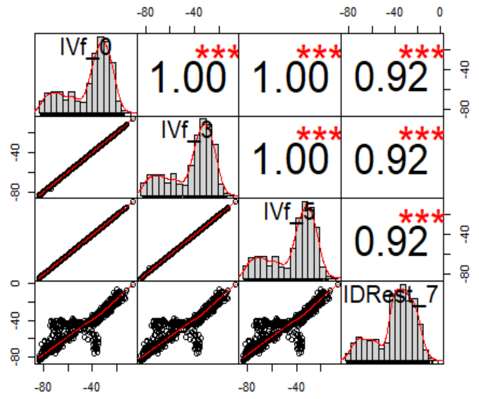

Voltage after stimulation:

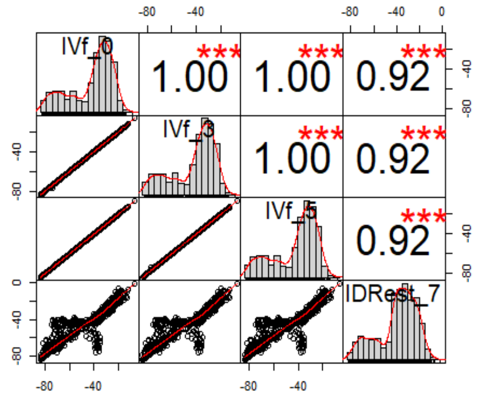

Spikecount:

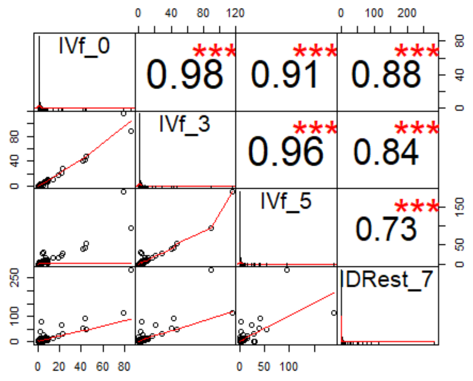

Features with missing values:

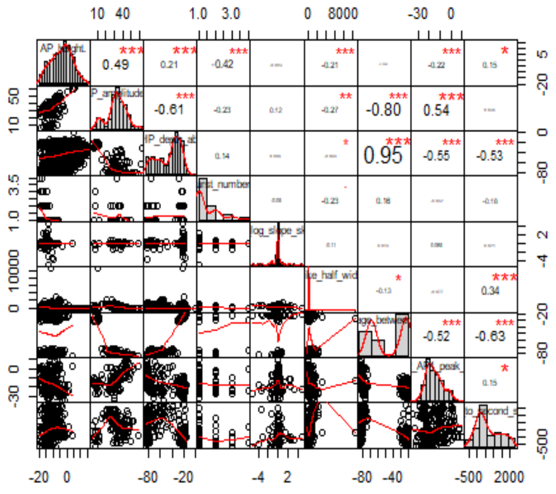

5. R packages

The results shown in this chapter were produced using the following packages:

The **tgp** package was used to apply the GP, GPllm, TGP and TGPllm models. In general, this package is designed for building surrogates of both stationary and nonstationary simulators, which can be either deterministic or noisy. It implements Bayesian techniques like the Metropolis-Hastings algorithm using a GP model for emulating the stationary components of the process. The GP model includes a nugget parameter estimated along with other parameters. The recent version of the **tgp** package facilitates the emulation of deterministic simulators by removing the nugget parameter from the model, supports design of experiments and includes one-dimensional and two-dimensional plots, which include tree drawing functions and higher dimension projection and slice options (Gramacy, 2007).[2]

The **laGP** package was used to apply a local approximate GP model (Gramacy, 2016).[3] The **randomForest** package used to apply Breiman and Cutler's Random Forests regression models with different number of trees (N_{tree}) and number of features per tree ($mtry$) (Liaw & Wiener, 2002). The **neuralnet** package was used to apply standard Neural Network models (Fritsch et al., 2016). The **caret** package was used in order to tune the RF and NN models hyperparameters (Kuhn, 2008). The tuning method bases its search for the optimal hyperparameters in terms of accuracy and Cohen's kappa for classification problems, and in terms of RMSE for regression problem.

PerformanceAnalytics is the R package that is the basis for all of the correlation plots in the preliminary statistical analysis in section 5.1 (Peterson et al., 2018). The main diagonal of these plots shows the histogram of each selected parameter/feature, the lower panel of the correlation graphs contains pairs plots of the selected parameters/features, and the upper panel shows Pearson's correlation coefficients for each of these pairs. All other plots in this paper were drawn with **ggplot2** (Wickham, 2011) and **ggpubr** (Kassambara, 2017)**.**

[2]https://doi.org/10.18637/jss.v019.i09
[3]https://doi.org/10.18637/jss.v072.i01

6. Main Effect Plots

This section presents examples of main effect plots for some of the input parameters and output features analyzed in Section 4. Note that each panel is scaled to match the strength of the effect, so that scales are not constant across panels.

Figure S2 - Bayesian sensitivity main effect: AP amplitude (parameters 1-5, generation 6)

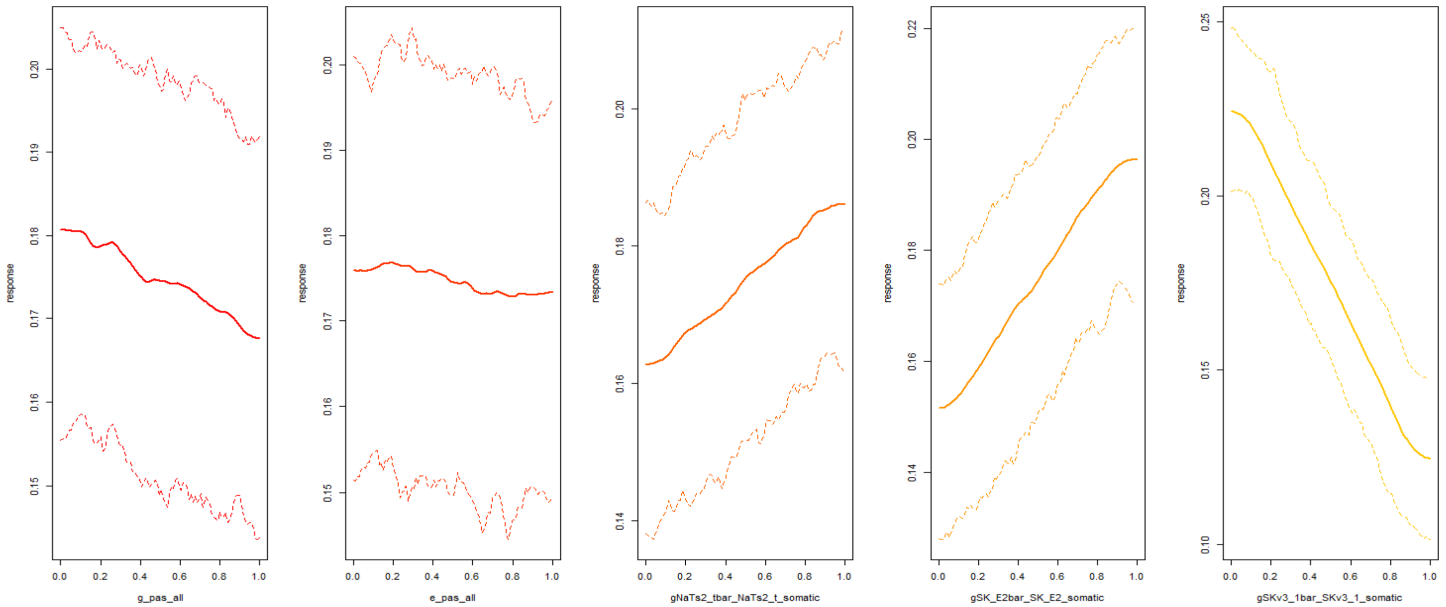

Figure S3 - Bayesian sensitivity main effect: AP amplitude (parameters 24-29, generation 6)

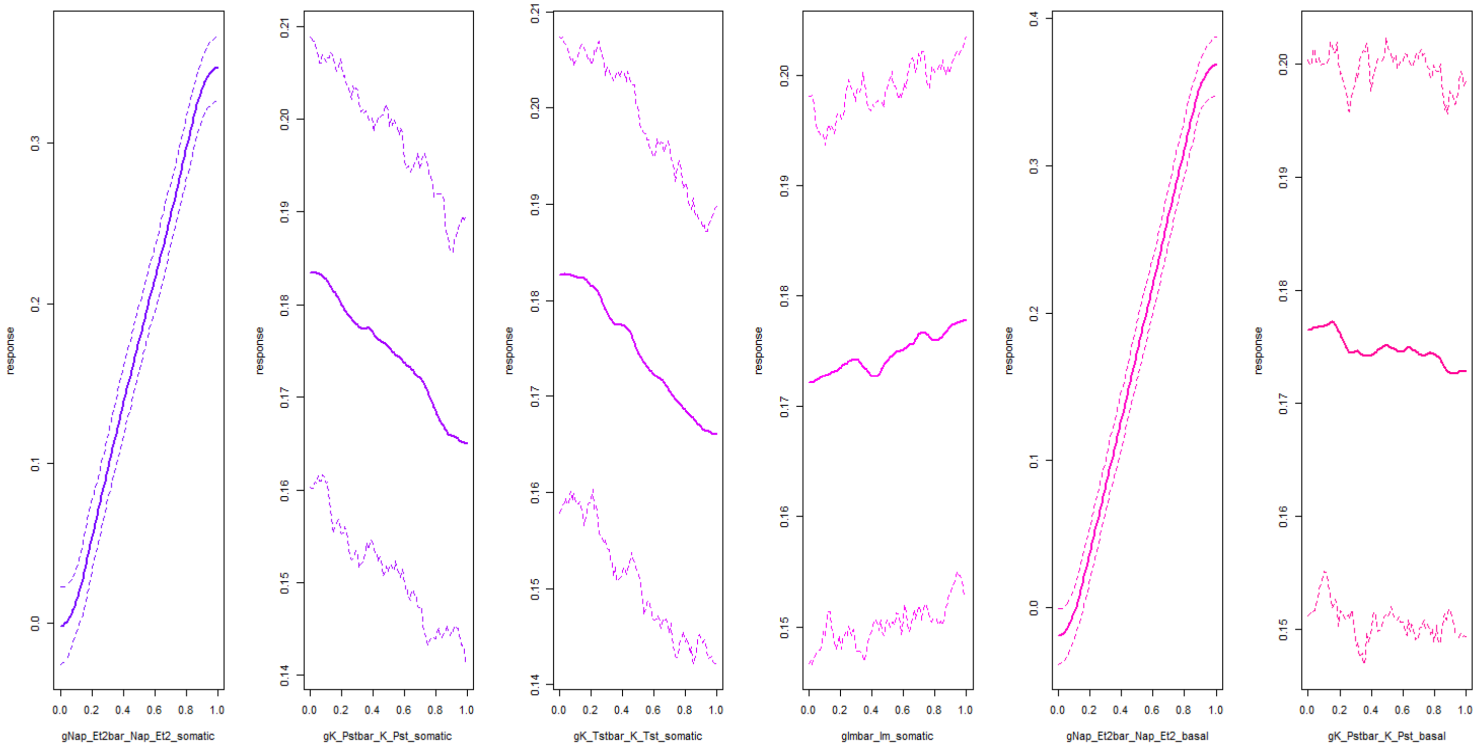

Figure S4 - Bayesian sensitivity main effect: minimum voltage between spikes (parameters 1-5, generation 6)

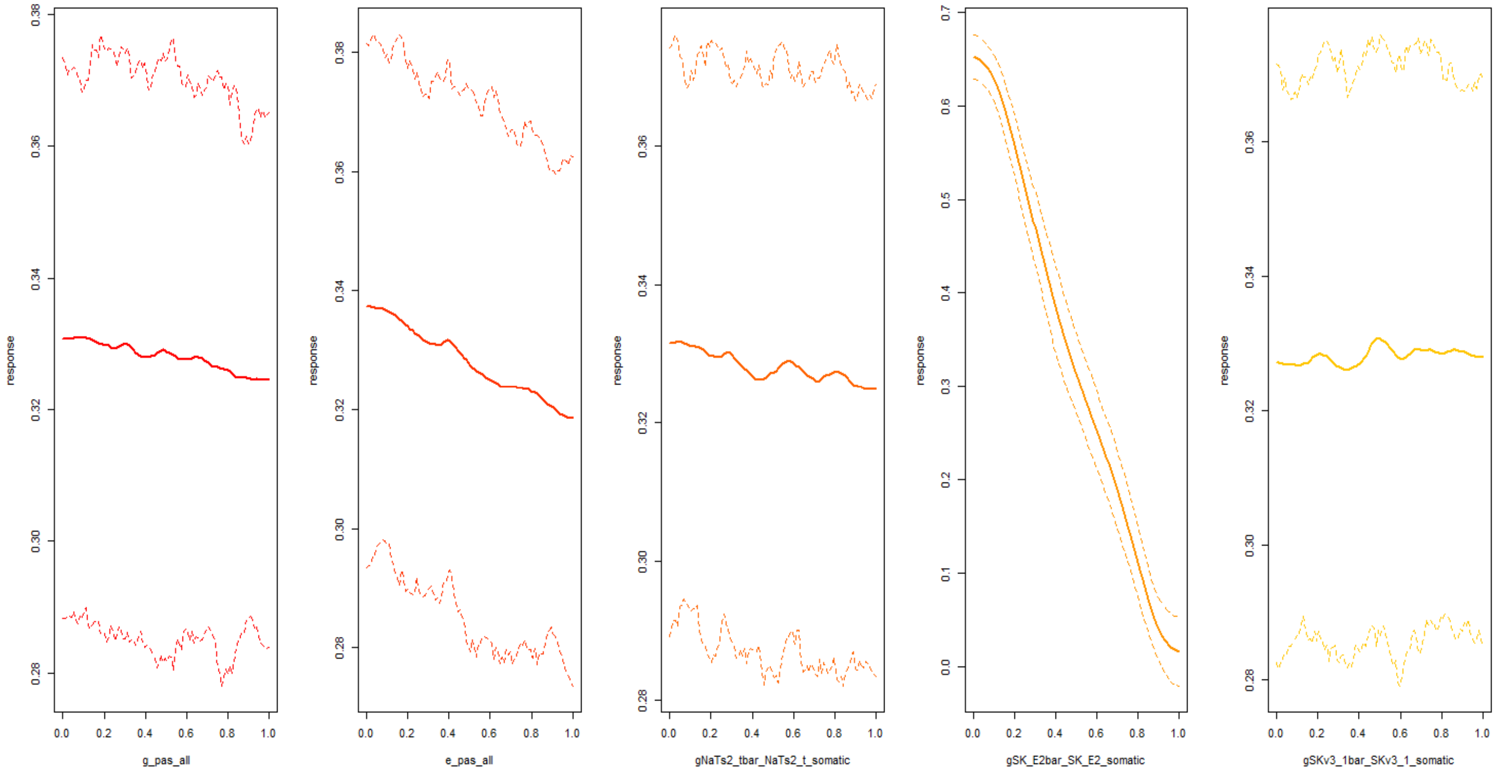

Figure S5 - Bayesian Sensitivity Main Effect: minimum voltage between spikes (parameters 24-29, generation 6)

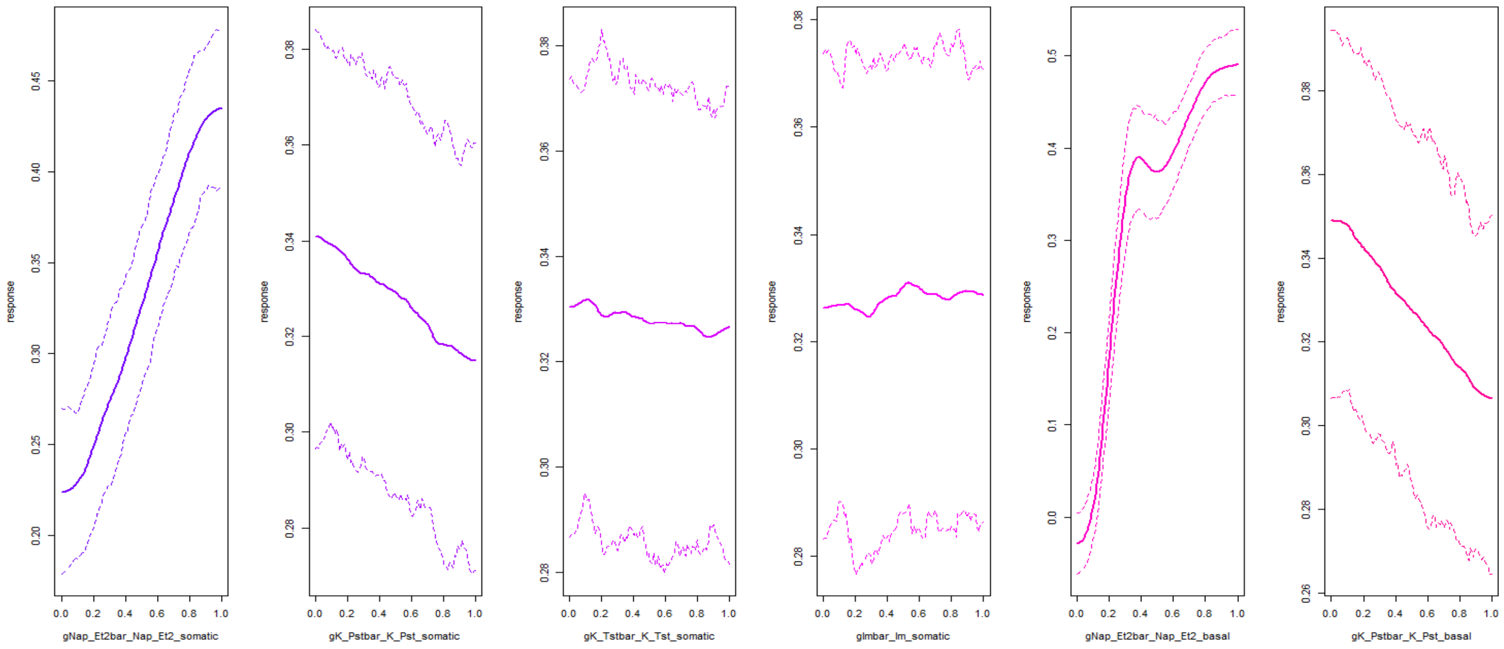

7. Bibliography

Chipman, H. A., George, E. I., & McCulloch, R. E. (2002). Bayesian Treed Models. *Machine Learning*, *48*(1), 299–320.

Fritsch, S., Guenther, F., & Guenther, M. F. (2016). Package 'neuralnet.' *The Comprehensive R Archive Network*.

Gramacy, R. B. (2005). *Bayesian treed Gaussian process models*. University of California, Santa Cruz.

Gramacy, R. B. (2007). tgp: An R Package for Bayesian Nonstationary, Semiparametric Nonlinear Regression and Design by Treed Gaussian Process Models. *Journal of Statistical Software*, *19*(9).

Gramacy, R. B. (2016). laGP: Large-Scale Spatial Modeling via Local Approximate Gaussian Processes in R. *Journal of Statistical Software*, *72*(1), 1–46.

Gramacy, R. B. (2020). *Surrogates: Gaussian Process Modeling, Design, and Optimization for the Applied Sciences*.

Gramacy, R. B., & Lee, H. K. H. (2008). *Gaussian Processes and Limiting Linear Models*.

Kassambara, A. (2017). ggpubr:"ggplot2" based publication ready plots. *R Package Version 0.1*, *6*.

Kuhn, M. (2008). Building predictive models in R using the caret package. *Journal of Statistical Software*, *28*(5), 1–26.

Liaw, A., & Wiener, M. (2002). Classification and regression by randomForest. *R News*, *2*(3), 18–22.

Peterson, B. G., Carl, P., Boudt, K., Bennett, R., Ulrich, J., Zivot, E., Cornilly, D., Hung, E., Lestel, M., & Balkissoon, K. (2018). Package 'PerformanceAnalytics.' *R Team Cooperation*.

Sobol, I. M. (1993). Sensitivity estimates for nonlinear mathematical models. *Mathematical Modelling and Computational Experiments*, *1*(4), 407–414.

Sobol, I. M. (2001). Global sensitivity indices for nonlinear mathematical models and their Monte Carlo estimates. *Mathematics and Computers in Simulation*, *55*(1–3), 271–280.

Wickham, H. (2011). ggplot2. *Wiley Interdisciplinary Reviews: Computational Statistics*, *3*(2), 180–185.
